# Long-term hospitalisations in survivors of paediatric solid tumours in France

**DOI:** 10.1038/s41598-022-22689-w

**Published:** 2022-10-27

**Authors:** Daniel Bejarano-Quisoboni, Nathalie Pelletier-Fleury, Rodrigue S. Allodji, Brice Fresneau, Majorie Boussac, Hélène Pacquement, François Doz, Delphine Berchery, Claire Pluchart, Piere-Yves Bondiau, Julie Nys, Angela Jackson, Charlotte Demoor-Goldschmidt, Agnes Dumas, Cécile Thomas-Teinturier, Boris Schwartz, Neige Journy, Carole Rubino, Giao Vu-Bezin, Dominique Valteau-Couanet, Chiraz El-Fayech, Christelle Dufour, Nadia Haddy, Florent de Vathaire

**Affiliations:** 1 Radiation Epidemiology Team, CESP, Inserm U1018, Villejuif, France; 2 Primary care and Prevention Team, CESP, Inserm U1018, Villejuif, France; 3 Université Paris-Saclay, UVSQ, Inserm, CESP, 94807 Villejuif, France; 4 Department of Research, Gustave Roussy, Villejuif, France; 5 Department of Children and Adolescent Oncology, Gustave Roussy, Villejuif, France; 6 French National Health Insurance (CNAM), Paris, France; 7 SIREDO Center (Care, Research, Innovation in Pediatric, Adolescents and Young Adults Oncology), Institut Curie, Paris, France; 8 University Paris Cité, Paris, France; 9 Epidemiology Unit, Claudius Regaud Institute, Toulouse, France; 10 Centre Hospitalier Universitaire de Reims, Pediatric Oncology, Reims, France; 11 Radiation Therapy, Antoine Lacassagne Cancer Center, Nice, France; 12 Pediatric Oncology, CHU Angers, Angers, France; 13 Department of Radiotherapy and Department of Supportive Care, François Baclesse Center, Caen, France; 14 Université Paris Cité, Inserm, ECEVE, UMR 1123, Paris, France; 15 Bicêtre Hospital, Service d’Endocrinologie et Diabétologie Pédiatrique AP-HP Université Paris Saclay, Paris, France

**Keywords:** Paediatric cancer, Health services

## Abstract

The late effects of treatments for childhood cancers may lead to severe and multiple health conditions requiring hospitalisation. We aimed to estimate the hospitalisation rate among childhood cancer survivors (CCS) in France, to compare them with the general population and to investigate the associated factors. We matched total of 5439 5-year solid CCS diagnosed before the age of 21 between 1945 and 2000 by sex, birth year and region of residence to 386,073 individuals of the French general population. After linkage with the national hospital discharge database, we estimated the relative hospitalisation rate (RHR), the absolute excess risks (AERs) and the relative bed-day ratio (RBDR) during 2006–2018. We used generalised linear models to estimate associations between hospitalisation and survivor characteristics. Overall, the RHR was 2.49 (95% confidence interval [CI] 2.46–2.52) and the RBDR was 3.49 (95% CI 3.46–3.51). We found that neoplasm-related hospitalisations had the highest AER (105.8 per 1000 person-years), followed by genitourinary system diseases (34.4 per 1000 person-years) and cardiovascular diseases (19.2 per 1000 person-years). In adjusted analysis, CCS treated with chemotherapy (risk ratio [RR] 1.62, 95% CI 1.53–1.70), radiotherapy (RR 2.11, 95% CI 1.99–2.24) or both (RR 2.59, 95% CI 2.46–2.73) had a higher risk of hospitalisation than the ones who had not received any of these treatments. CCS treated during the past decades by chemotherapy and/or radiotherapy now had a higher hospitalisation risk for all main categories of diagnosis than the general population. Prevention strategies and medical surveillance programmes may promote a long-term decrease in the hospitalisation rate among CSS.

## Introduction

Advances in cancer treatment, such as improvements in chemotherapy regimens, radiation techniques and surgery, have allowed achieving 5-year survival rates of more than 80% in patients with paediatric cancer. However, late effects from cancer therapies continue to be a challenge. It is estimated that two thirds of childhood cancer survivors (CCS) will experience at least one treatment-related adverse event and 40% will experience at least one severe or life-threatening or disabling event several years after the cancer diagnosis^1,2^. Potential life-threatening or disabling late effects include second neoplasms, cardiovascular diseases, growth problems and mental health issues^3^. This increased morbidity may lead to the development of complex health conditions requiring hospitalisation^4^.

Some researchers have evaluated the long-term risk of hospitalisation among CCS and have reported an overall increased risk in survivors compared with the general population^4–9^. In addition, their average length of stay in the hospital was up to 35% longer than patients without a history of cancer^10^. A better understanding of the long-term hospitalisation of CCS is thus important to evaluate their health conditions and health care–related costs several years after cure.

In France, there are about 50,000 adult CCS, with a growing number of long-term survivors^11^. However, no studies have analysed and detailed the hospitalisation rate in long-term CCS. The aim of this paper was to estimate the hospitalisation rate among CCS residing in France compared those of the French general population. We have also described the hospitalisation-related clinical diagnoses and have investigated cancer-related factors associated with an increased probability of hospitalisation.

## Materials and methods

### Study population

The French Childhood Cancer Survivor Study (FCCSS) is a retrospective cohort of 7670 5-year CCS diagnosed for solid cancer or lymphoma (all malignancies except leukaemia) before the age of 21 years between 1945 and 2000 in five cancer centres in France. Cancer diagnoses were classified according to ICCC-3, with the exception of thyroid cancer, which was included in a separate group due to the specificity of iodine treatment and its consequences. Detailed information on the methods for data collection and patients has been already described in several articles^12,13^. To study the FCCSS hospitalisation records, we selected survivors who were alive in January 2006, living in metropolitan France and who were linked to the National Health Data System (French acronym: SNDS). Of the 7670 5-years survivors from the FCCSS, 6,818 were still alive on 1 January 2006.

### Data sources

The SNDS is the health care claims dataset in France; it covers around 99% of the population^14^. It contains exhaustive data (beginning in 2006) on billing and reimbursement of beneficiaries, including private and public hospital data collected in the national hospital discharge database (French acronym: PMSI).

The PMSI is divided into four categories corresponding to hospitalisation in conventional hospital units (short stays), homecare units, rehabilitation and psychiatry institutions. It includes some demographic characteristics of patients (age, gender, place of residence) as well as clinical information of the hospitalisations such as the in-patient bed-days, primary and secondary diagnoses according to the 10th revision of the International Classification of Diseases (ICD-10), procedures performed, medications administered and the dates of death and cost information under the diagnosis‐related group system^15^.

Linkage and access of SNDS data of FCCSS cohort was provided by the national health insurance fund (French acronym: CNAM) by probabilistic matching using the survivor's family and first names, sex, date and place of birth and unique arbitrary number. The percentage of survivors linked to SNDS data after this procedure was 81.9% (n = 5583/6818), with data availability from 2006 to 2018.

### Reference sample

We obtained a reference sample from the General Sample of Beneficiaries (French acronym: EGB). The EGB is a 1/97th anonymised random permanent sample of all population included in the SNDS (n ≈ 830,000 in 2021) which has been shown to be representative of the French general population^16^. Health care claims including hospitalisation records (PMSI) are available in the EGB; however, information regarding hospitalisation in rehabilitation and psychiatric institutions has not yet been supplied. The reference sample was selected matched by sex, year of birth and the region (French administrative area) of residence and randomly assigned to each FCCSS survivors with the same characteristics.

### Hospitalisation measures

Using the PMSI, we obtained hospitalisation records in conventional hospital units from January 2006 to December 2018 or death—whichever came first—for each CCS and individual of the reference population. Our endpoints of interest were (1) the total number of hospitalisations and (2) the total number of bed-days spent in hospital, which is the number of days in which the patient stays overnight in a hospital, excluding day hospital visits. We grouped hospitalisations according to the primary diagnosis into the 19 main groups of the ICD-10, excluding the following categories: pregnancy and childbirth (O00–O99), certain conditions originating in the perinatal period (P00–P96), external causes of morbidity and mortality (V01–Y98) and codes for special purposes (U00–U99), in order to focus on hospitalisation potentially linked to childhood cancer sequels.

### Ethics approval

The study was approved by the French Data Protection Authority (French acronym: CNIL) (Authorization n°902287) and by the ethics committee of the French National Institute of Health and Medical Research (French acronym: INSERM). Informed consent was obtained for patients who could be contacted (n = 3312), and posters containing information about the study and how to decline participation were displayed in the French paediatric oncology departments. Finally, we obtained a specific act in law from the French ‘Conseil d’Etat’, the highest court in France (Order 2014-96 of 2014 February 3), that approved the cession of the SNDS data for all patients included in the FCCSS with or without informed consent. All methods were performed in accordance with the relevant guidelines and regulations.

### Statistical analysis

The hospitalisation and bed-day rates were calculated for both the FCCSS and reference populations as the total number of hospitalisations or bed-days divided by the number of person-years (PY) at risk and expressed as the rate per 1000 PY. Time during hospitalisation was not counted as time at risk when calculating the hospitalisation rate. To compare the FCCSS hospitalisation rate to the reference population, we calculated: (1) the absolute excess risk (AER) as the difference between the hospitalisation or bed-day rate of the FCCSS and reference populations expressed per 1000 PY, and (2) the relative hospitalisation rate (RHR) and the relative bed-day ratio (RBDR) with their corresponding 95% confidence intervals (CIs) as the division of the hospitalisation or bed-day rate of the FCCSS and reference populations based on the assumption that the observed number of hospitalisations and bed-days followed a Poisson distribution. We calculated the AER, RHR and RBDR for overall hospitalisations and for each ICD-10 main group.

We used generalised linear models (GLMs) to model the number of hospitalisations and bed-days. We used the expected number of hospitalisations or bed-days as an offset to study the risk for FCCSS survivors relative to that for the reference population. We adjusted the models for sex, year of cancer diagnosis, age at cancer diagnosis, age in 2006, type of primary cancer, age and cancer treatment(s) received (i.e. chemotherapy, radiotherapy, and/or surgery). We chose neuroblastoma as the reference primary cancer variable because it is one of the larger groups of cancer with the same histology. We have reported risk estimates as risk ratios (RRs) and 95% CIs. Finally, we executed separate models for each ICD main group to evaluate risk factors for the different types of hospitalisations. We performed statistical analyses by using SAS 9.4 software (SAS Institute, Cary, NC, USA), considering p < 0.05 to be significant.

## Results

We included 5439 FCCSS survivors and 386,073 reference persons. Each CCS was assigned an average of 71 (SD: 73.9) unique reference persons. Between 1 January 2006 and 31 December 2018, 3756 CCS (69%) and 208,217 reference persons (54%) had had at least one hospitalisation. By the end of the follow-up, 383 (7%) FCCSS survivors and 38,458 (10%) reference persons had died. About 28% of FCCSS patients were less than 20 years old at the start of the SNDS follow-up in 2006, 10% being 40 years old or more (Table 1). The average delay between childhood cancer treatment and 2006 was 19.9 years (SD: 9.9, interquartile range 12–26).

**Table 1:** Survivor characteristics and hospitalisation and bed-day rates.

	Total Patients (%)	Patients Hospitalized (%)	N° Hospitalizations	Hospitalization rate in FCCSS (per 1000 PY)	Hospitalization rate in EGB (per 1,000 PY)	AER per 1,000 PY	RHR (95% CI)	N° Bed-days	Bed-days rate in FCCS (per 1000 PY)	Bed-days rate in EGB (per 1,000 PY)	AER per 1000 PY	RBDR (95% CI)
All	5439	3756	27,598	401.2	161.3	240.0	2.49 (2.46–2.52)	74,814	1084.4	311.1	773.3	3.49 (3.46–3.51)
**Sex**												
Man	2970 (54.6)	1982 (52.8)	13,646	363.5	145.4	218.1	2.5 (2.46–2.54)	35,870	952.9	313.6	639.3	3.04 (3.01–3.07)
Women	2469 (45.4)	1774 (47.2)	13,952	446.6	177.4	269.2	2.52 (2.48–2.56)	38,944	1242.3	308.6	933.7	4.03 (3.99–4.07)
**Age at January 2006 (Start date)**												
< 20	1535 (28.2)	783 (20.8)	4012	202.0	85.9	116.1	2.35 (2.28–2.43)	7755	390.0	136.3	253.7	2.86 (2.8–2.93)
20–30	2066 (38)	1488 (39.6)	10,665	407.1	111.4	295.7	3.66 (3.59–3.73)	27,159	1033.7	182.7	851.0	5.66 (5.59–5.73)
31–40	1311 (24.1)	1038 (27.6)	8125	496.0	165.8	330.2	2.99 (2.93–3.06)	23,168	1408.9	304.5	1104.4	4.63 (4.57–4.69)
> = 41	527 (9.7)	447 (11.9)	4796	756.5	287.0	469.5	2.64 (2.56–2.71)	16,732	2620.3	630.7	1989.6	4.15 (4.09–4.22)
**Status at December 2018 (Ending date)**												
Alive	5056 (93)	3389 (90.2)	19,606	298.9	163.7	135.2	1.83 (1.8–1.85)	44,789	681.5	310.8	370.7	2.19 (2.17–2.21)
Death	383 (7)	367 (9.8)	7992	2509.9	139.4	2370.5	18 (17.61–18.4)	30,025	9192.1	314.3	8877.8	29.25 (28.92–29.58)
**Year of diagnosis**												
< 1970	351 (6.5)	300 (8)	2925	688.3	300.9	387.4	2.29 (2.21–2.37)	10,688	2498.0	677.7	1820.3	3.69 (3.62–3.76)
1970–1979	980 (18)	783 (20.8)	7387	611.4	197.1	414.3	3.1 (3.03–3.17)	19,820	1633.2	375.3	1257.9	4.35 (4.29–4.41)
1980–1989	1809 (33.3)	1335 (35.5)	9905	433.3	129.5	303.8	3.34 (3.28–3.41)	29,690	1294.1	227.8	1066.3	5.68 (5.62–5.75)
> = 1990	2299 (42.3)	1338 (35.6)	7381	249.4	93.0	156.4	2.68 (2.62–2.74)	14,616	493.2	150.3	342.9	3.28 (3.23–3.33)
**Age at first cancer**												
0–1	1288 (23.7)	770 (20.5)	5687	345.1	126.1	219.0	2.74 (2.67–2.81)	12,031	728.7	227.3	501.4	3.21 (3.15–3.26)
2–4	1276 (23.5)	857 (22.8)	5864	362.9	146.6	216.3	2.48 (2.41–2.54)	18,753	1156.7	268.3	888.4	4.31 (4.25–4.37)
5–9	1207 (22.2)	868 (23.1)	6617	437.5	164.5	273.0	2.66 (2.6–2.73)	19,619	1292.5	326.9	965.6	3.95 (3.9–4.01)
10–14	1108 (20.4)	839 (22.3)	6839	492.1	193.7	298.4	2.54 (2.48–2.6)	18,031	1292.9	397.4	895.5	3.25 (3.21–3.3)
≥ 15	560 (10.3)	422 (11.2)	2591	363.5	199.3	164.2	1.82 (1.75–1.9)	6380	893.0	384.3	508.7	2.32 (2.27–2.38)
**First primary cancer type**												
Other solid cancer	312 (5.7)	222 (5.9)	1622	414.1	152.8	261.3	2.71 (2.58–2.84)	3645	928.2	285.9	642.3	3.25 (3.14–3.35)
Kidney tumors	825 (15.2)	551 (14.7)	4712	453.1	172.1	281.0	2.63 (2.56–2.71)	12,249	1174.0	335.7	838.3	3.5 (3.44–3.56)
Neuroblastoma	746 (13.7)	466 (12.4)	3124	327.1	126.7	200.4	2.58 (2.49–2.67)	6727	703.1	219.9	483.2	3.2 (3.12–3.27)
Lymphoma	931 (17.1)	669 (17.8)	4990	421.7	178.6	243.1	2.36 (2.3–2.43)	12,512	1054.3	363.2	691.1	2.9 (2.85–2.95)
Soft tissue sarcomas	591 (10.9)	417 (11.1)	3098	410.7	184.1	226.6	2.23 (2.15–2.31)	7680	1015.3	360.3	655.0	2.82 (2.76–2.88)
Bone sarcomas	476 (8.8)	368 (9.8)	2428	400.9	187.0	213.9	2.14 (2.06–2.23)	6129	1009.2	371.8	637.4	2.71 (2.65–2.78)
Central nervous system tumor	708 (13)	567 (15.1)	4314	502.1	141.0	361.1	3.56 (3.45–3.67)	15,558	1801.7	286.3	1515.4	6.29 (6.2–6.39)
Gonadal/Germ cell tumours	334 (6.1)	231 (6.2)	1441	337.2	182.7	154.5	1.85 (1.75–1.94)	4731	1103.7	352.2	751.5	3.13 (3.04–3.22)
Thyroid tumor	48 (0.9)	33 (0.9)	150	247.0	303.8	-56.8	0.81 (0.69–0.95)	424	696.8	595.4	101.4	1.17 (1.06–1.29)
Retinoblastoma	468 (8.6)	232 (6.2)	1719	285.8	105.0	180.8	2.72 (2.59–2.85)	5159	855.8	165.7	690.1	5.17 (5.03–5.31)
**Treatment received**												
No radiotherapy or chemotherapy	720 (13.2)	394 (10.5)	1789	192.6	152.0	40.6	1.27 (1.21–1.33)	3105	333.9	280.8	53.1	1.19 (1.15–1.23)
Radiotherapy	696 (12.8)	549 (14.6)	4670	549.3	233.6	315.7	2.35 (2.28–2.42)	15,418	1804.4	500.9	1303.5	3.60 (3.55–3.66)
Chemotherapy	1990 (36.6)	1236 (32.9)	7275	284.5	125.8	158.7	2.26 (2.21–2.31)	19,361	755.6	226.9	528.7	3.33 (3.28–3.38)
Radiotherapy and Chemotherapy	2033 (37.4)	1577 (42)	13,864	545.3	158.3	387.0	3.45 (3.39–3.5)	36,930	1446.8	297.7	1149.1	4.86 (4.81–4.91)

FCCSS, French Childhood Cancer Survivor Study; EGB, general sample of beneficiaries; AER, absolute access risk; PY, person-year; RHR, relative hospitalization ratio; RBDR, relative bed-days ratios.

### Total number of hospitalisations and bed-days

The following results are presented in Table 2. We identified 27,598 hospitalisations in FCCSS survivors, which accounted for 74,814 in-patient bed-days. For the FCCSS survivors, the hospitalisation rate was 401.2 per 1000 PY while the bed-day rate was 1084.4 per 1000 PY. In the matched reference population, the hospitalisation rate was 161.3 per 1000 PY and the bed-day rate was 311.1 per 1000 PY. Hence, the AER of hospitalisation was 239.9 per 1000 PY and the AER of in-patient bed-days was 773.2 per 1000 PY for FCCSS survivors. The RHR was 2.49 (95% CI 2.46–2.52, p < 0.001), meaning that FCCSS survivors were hospitalised more than twice as often as the matched reference population. Additionally, they had more than three times as many in-patient bed-days as the reference population (RBDR 3.49, 95% CI 3.46–3.51, p < 0.001). When excluding hospitalisation for neoplasms, which could be linked to long-term relapses of childhood cancer and secondary neoplasms, these numbers were, respectively, RHR = 2.12 (95% CI 2.08–2.15) and RBDR = 3.36 (95% CI 3.33–3.38).

**Table 2:** Hospitalisations and bed-days in the French Childhood Cancer Survivor Study (FCCSS) survivors and the reference sample according to the 10th revision of the International Classification of Diseases.

	Hospitalizations							Bed-days in the hospital						
	N° Hospitalizations in FCCSS	Hospitalization rate in FCCSS (per 1000 PY)	N° Hospitalizations in EGB	Hospitalization rate in EGB (per 1000 PY)	AER per 1000 PY	RHR	(95% CI)	N° Bed-Days in FCCSS	Bed-Days rate in FCCS (per 1000 PY)	N° Bed-Days in EGB	Bed-Days rate in EGB (per 1000 PY)	AER per 1000 PY	RBDR	(95% CI)
Total	27,598	401.2	805,758	161.3	240.0	2.49	(2.46–2.52)	74,814	1084.4	1,555,993	311.1	773.3	3.49	(3.46–3.51)
Infections	245	3.6	7585	1.5	2.0	2.35	(2.06–2.66)	1512	21.9	35,350	7.1	14.8	3.10	(2.95–3.26)
Neoplasms	10,100	146.8	205,097	41.0	105.8	3.58	(3.51–3.65)	16,156	234.2	289,619	57.9	176.3	4.04	(3.98–4.11)
Haematological	237	3.4	5233	1.0	2.4	3.29	(2.88–3.74)	858	12.4	19,169	3.8	8.6	3.24	(3.03–3.47)
Endocrine	830	12.1	17,153	3.4	8.6	3.51	(3.28–3.76)	2185	31.7	67,426	13.5	18.2	2.35	(2.25–2.45)
Mental	300	4.4	18,899	3.8	0.6	1.15	(1.03–1.29)	782	11.3	48,923	9.8	1.6	1.16	(1.08–1.24)
Neurological	803	11.7	23,414	4.7	7.0	2.49	(2.32–2.67)	4433	64.3	55,985	11.2	53.1	5.74	(5.57–5.91)
Ocular	326	4.7	13,992	2.8	1.9	1.69	(1.51–1.89)	453	6.6	8277	1.7	4.9	3.97	(3.61–4.35)
Auditory	109	1.6	4311	0.9	0.7	1.84	(1.51–2.22)	236	3.4	6865	1.4	2.0	2.49	(2.18–2.83)
Cardiovascular	1900	27.6	42,035	8.4	19.2	3.28	(3.14–3.43)	9065	131.4	139,661	27.9	103.5	4.70	(4.61–4.8)
Pulmonary	601	8.7	20,994	4.2	4.5	2.08	(1.92–2.25)	4155	60.2	84,894	17.0	43.2	3.55	(3.44–3.66)
Gastrointestinal	2113	30.7	116,056	23.2	7.5	1.32	(1.27–1.38)	5901	85.5	171,616	34.3	51.2	2.49	(2.43–2.56)
Skin	393	5.7	14,316	2.9	2.8	1.99	(1.8–2.2)	872	12.6	25,460	5.1	7.5	2.48	(2.32–2.65)
Musculoskeletal	881	12.8	54,634	10.9	1.9	1.17	(1.1–1.25)	3317	48.1	137,421	27.5	20.6	1.75	(1.69–1.81)
Genitourinary	3462	50.3	79,401	15.9	34.4	3.17	(3.06–3.27)	11,259	163.2	148,360	29.7	133.5	5.50	(5.4–5.6)
Congenital Malformations	153	2.2	3514	0.7	1.5	3.16	(2.68–3.71)	498	7.2	7390	1.5	5.7	4.88	(4.47–5.33)
Symptoms Unclassified	1160	16.9	42,017	8.4	8.5	2.01	(1.89–2.12)	3108	45.0	60,888	12.2	32.9	3.70	(3.57–3.83)
Injury—Poisoning	1003	14.6	53,385	10.7	3.9	1.36	(1.28–1.45)	4344	63.0	136,490	27.3	35.7	2.31	(2.24–2.38)
Other Factors	2982	43.4	83,722	16.8	26.6	2.59	(2.5–2.68)	5680	82.3	112,199	22.4	59.9	3.67	(3.57–3.77)
Total Excluding Neoplasms	17,498	254.4	600,661	120.2	134.2	2.12	(2.08–2.15)	58,658	850.2	1,266,374	253.2	597.0	3.36	(3.33–3.38)

FCCSS, French Childhood Cancer Survivor Study; EGB, general sample of beneficiaries; AER, absolute access risk; PY, person-year; RHR, relative hospitalization ratio; RBDR, relative bed-days ratios.

### Hospitalisations and bed-days by the main diagnostic groups

FCCSS survivors were more frequently hospitalised and had more in-patient bed-days than the matched reference population for all diagnostic groups. Neoplasm-related hospitalisation had the highest AER (105.8 per 1000 PY), followed by genitourinary system diseases (34.4 per 1000 PY), factors influencing health status and contact with health services (other factors) (26.6 per 1000 PY) and circulatory system diseases (19.2 per 1000 PY). There were the fewest hospitalisations for mental and behavioural disorders and auditory issues (AER = 0.6 and 0.7 per 1000 PY, respectively) (Table 2). Details of the main diagnoses of the hospitalisations are reported in Supplementary Table 1.

**Figure 1:**
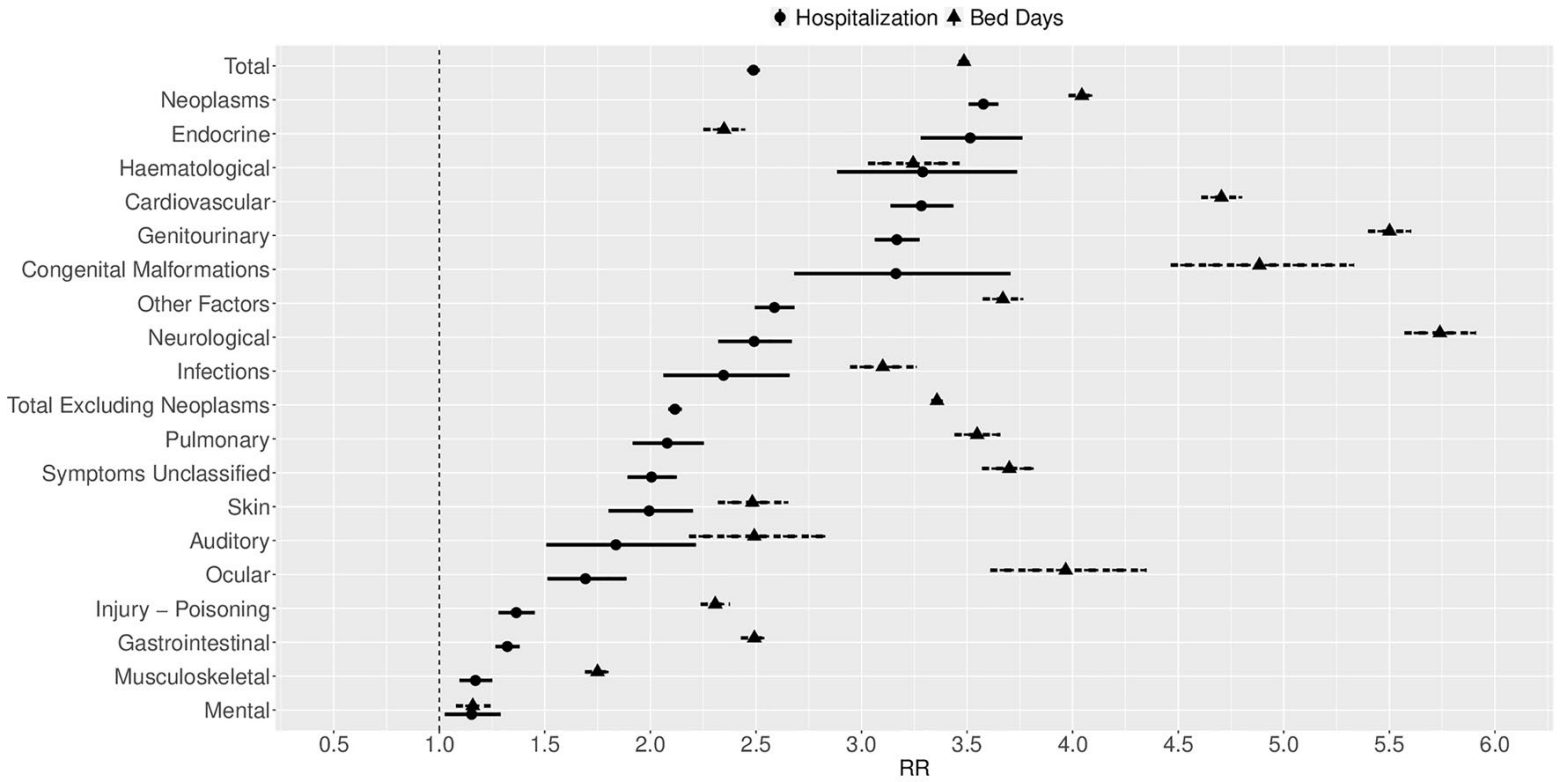
The relative hospitalisation ratio and the relative bed-day ratio according to the 10th revision of the International Classification of Diseases.

As a general matter, hospitalisations were not only more frequent in FCCSS survivors than in matched reference population, but their stays were longer for each diagnostic group. The AER of bed-days was higher than the AER of hospitalisation in all main diagnostic groups. In addition, the RBDR was higher than the RHR for all diagnostic groups except endocrine and haematological diseases (Fig. 1, Table 2). This phenomenon was particularly pronounced for hospitalisation related to nervous system and genitourinary system pathologies, leading to over five times as many in-patient bed-days in FCCSS survivors as the reference population (RBDR 5.74, 95% CI 5.57–5.91 and RBDR 5.50, 95% CI 5.40–5.60, respectively).

### Hospitalisations and bed-days and the survivors’ characteristics

**Figure 2:**
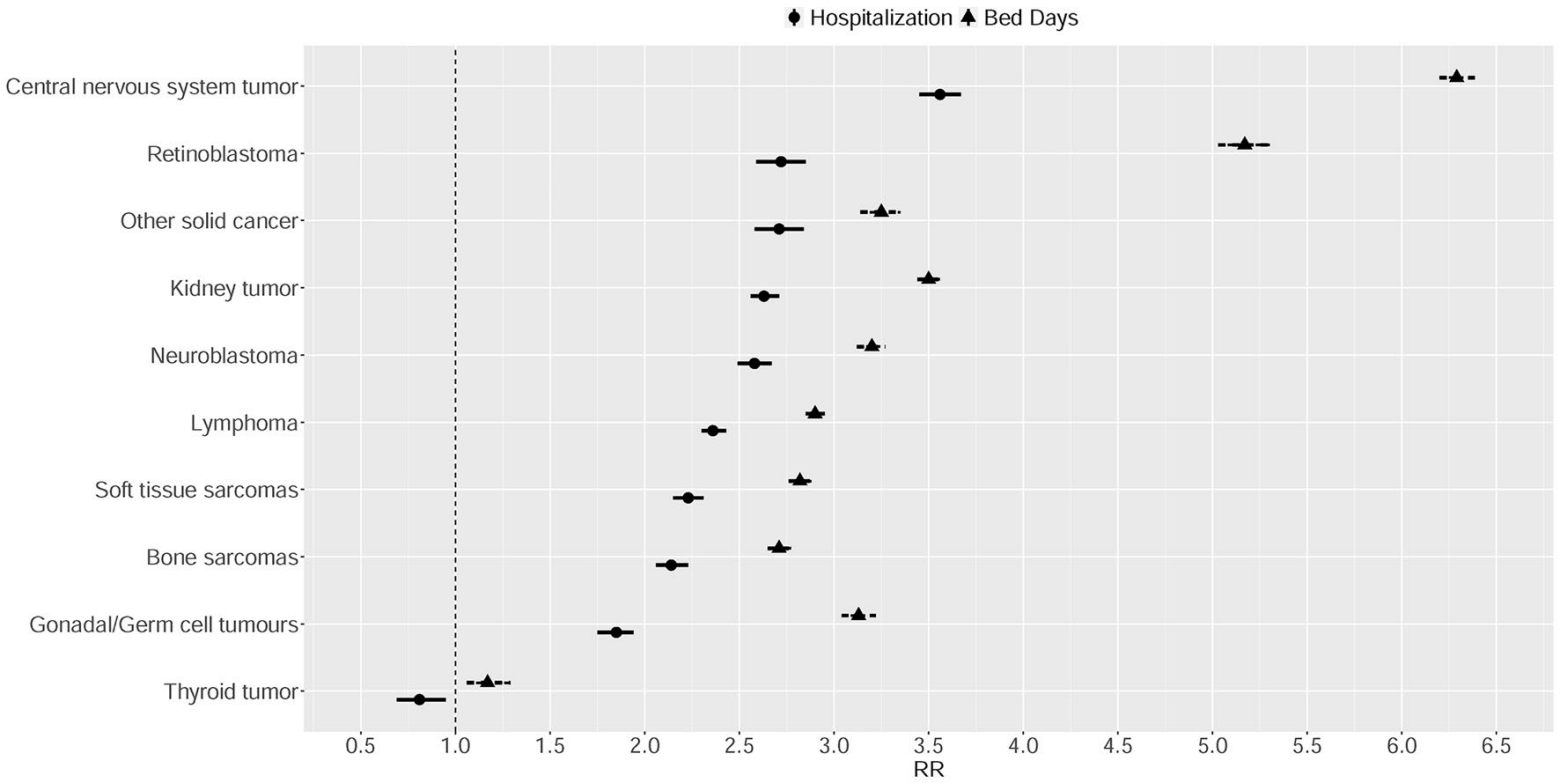
The relative hospitalisation ratio and the relative bed-day ratio by the type of primary cancer.

Figure 2 shows that the hospitalisation rate was significantly higher in all types of primary cancer compared with the reference population, except in survivors of thyroid tumours (RHR 0.81, 95% CI 0.69–0.95) (Table 1). Central nervous system (CNS) tumour survivors had the highest RHR (3.56, 95% CI 3.45–3.67) and the highest RBDR (6.29, 95% CI 6.20–6.39) (Table 1). In detail, CNS tumour survivors were most likely to be hospitalised for congenital malformations (RHR 14.34, 95% CI 11.22–18.06), diseases of the nervous system (RHR 10.48, 95% CI 9.44–11.61) and endocrine-related diseases (RHR 10.45, 95% CI 9.29–11.7), with a very high in-patient bed-day rate for the first groups of pathologies (RBDR 33.88, 95% CI 30.27–37.81 and RBDR 32.19, 95% CI 31.02–33.39, respectively), but not for hospitalisation for endocrine diseases (Supplementary Table 1).

In a multivariate analysis, compared with neuroblastoma, thyroid tumour survivors were at lower risk of both hospitalisation and in-patient bed-days (RR 0.66, 95% CI 0.56–0.78 and RR 0.73, 95% CI 0.66–0.80, respectively), while CNS tumour survivors (RR 1.29, 95% CI 1.22–1.36), kidney tumour survivors (RR 1.09, 95% CI 1.04–1.14) and other primary cancer survivors (RR 1.39, 95% CI 1.30–1.48) were at higher risk (Table 3). The in-patient bed-day risk was higher among survivors of CNS tumours, gonadal tumours and retinoblastoma (Table 3).

**Table 3:** Multivariate analysis of the total number of hospitalizations and bed-days.

	Number of hospitalizations	Number of bed-days
	RR (95% CI)	RR (95% CI)
Intercept	1.79 (1.66–1.93)***	0.93 (0.89–0.98)***
Women (Ref = Men)	0.97 (0.95–0.99)***	1.29 (1.27–1.31)***
Age in 2006	0.99 (0.99–0.99)***	0.99 (0.98–0.99)***
**Age at first cancer (Ref = 0–1)**		
2–4	0.89 (0.86–0.93)***	1.38 (1.35–1.41)***
5–9	0.99 (0.94–1.03)	1.33 (1.29–1.37)***
10–14	1.04 (0.99–1.11)	1.23 (1.19–1.28)***
≥ 15	0.83 (0.77–0.9)***	1 (0.96–1.05)
**Year of diagnosis (Ref = > 1990)**		
< 1970	1.05 (0.93–1.19)	1.86 (1.73–2)***
1970–1979	1.32 (1.22–1.43)***	1.82 (1.74–1.9)***
1980–1989	1.34 (1.27–1.41)***	2.07 (2.01–2.13)***
**First primary cancer type (Ref = Neuroblastoma)**		
Other solid cancer	1.39 (1.3–1.48)***	1.4 (1.34–1.47)***
Kidney tumors	1.09 (1.04–1.14)***	0.98 (0.95–1.01)
Lymphoma	0.99 (0.94–1.04)	0.98 (0.95–1.01)
Soft tissue sarcomas	0.97 (0.92–1.03)	0.95 (0.91–0.98)***
Bone sarcomas	1.01 (0.95–1.08)	1.03 (0.99–1.07)
Central nervous system tumor	1.29 (1.22–1.36)***	1.97 (1.9–2.03)***
Gonadal/Germ cell tumours	1.02 (0.96–1.1)	1.39 (1.34–1.45)***
Thyroid tumor	0.66 (0.56–0.78)***	0.73 (0.66–0.8)***
Retinoblastoma	0.98 (0.92–1.04)	1.59 (1.54–1.65)***
**Treatment (Ref = No radiotherapy or chemotherapy)**		
Chemotherapy	1.62 (1.53–1.70)***	2.63 (2.53–2.74)***
Radiotherapy	2.10 (1.98–2.22)***	2.72 (2.61–2.83)***
Radiotherapy and Chemotherapy	2.60 (2.46–2.73)***	3.72 (3.58–3.86)***

RR, risk ratio; CI, confidence interval.

***p < 0.01, **p < 0.05.

FCCSS women were slightly more frequently hospitalised and accumulated more bed-days than men (Table 1). In an adjusted analysis, compared with the reference population, FCCSS women had a lower relative hospitalisation risk (RR 0.97, 95% CI 0.95–0.99) than FCCSS men; however, they had a higher in-patient relative bed-day risk (RR 1.29, 95% CI 1.27–1.31) (Table 3).

In a univariate analysis, there was no clear variation in the RHR according to the calendar period and the age at childhood cancer diagnosis, nor with the age at the start of the SNDS follow-up (2006) (Table 1). Compared with the reference population, the hospitalisation rate in the FCCSS survivors increased with age. This phenomenon was denoted by the higher AER with increasing age at the start of the SNDS follow-up. There were similar results for in-patient bed-days (Table 1). In a multivariate analysis, the variations in the adjusted RHR according to age at childhood cancer diagnosis were very low, and the variations in adjusted RBDR were low, whereas the RHR and RBDR significantly decreased as the age at the start of the SNDS follow-up increased. These changes were greater in patients treated between 1970 and 1990 than in the ones treated before that time (Table 3).

When investigating the role of age in each hospitalisation category for the RHR (Supplementary Table 2) and the RBDR (Supplementary Table 3) in a multivariate analysis, there were no clear variations, except for an increase with age at childhood cancer for hospitalisation for auditory diseases and a decrease for hospitalisation for genitourinary diseases.

### Role of treatments

Survivors who had been treated with surgery or who had not received treatment had a small increase in the hospitalisation rate (RHR 1.27, 95% CI 1.21–1.33) (Table 1). However, the hospitalisation and bed-day risks increased in survivors who had been treated with chemotherapy (RR 1.62, 95% CI 1.53–1.70 and RR 2.63, 95% CI 2.53–2.74, respectively), radiotherapy (RR 2.11, 95% CI 1.98–2.22 and RR 2.72, 95% CI 2.61–2.83, respectively) or both (RR 2.60, 95% CI 2.46–2.73 and RR 3.72, 95% CI 3.58–3.86, respectively) compared with survivors who had not received these treatments (Table 3). Chemotherapy was also associated with a significant increase in hospitalisation related to neoplasms, endocrine disorders and cardiovascular diseases; this increase was enhanced by radiotherapy (Supplementary Table 3). Chemotherapy was the most important risk factor for hospitalisation related to genitourinary system diseases (RHR 6.53, 95% CI 5.34–7.99) and blood disorders (RR 3.59, 95% CI 1.69–7.66) while radiotherapy was the most important determinant for hospitalisation due to nervous system diseases (RR 1.81, 95% CI 1.34–2.44) (Supplementary Table 3). There were similar results concerning childhood cancer treatments for the bed-day rate in the different hospitalisation groups (Supplementary Table 4).

## Discussion

In a cohort of 5439 5-year solid CCS, we found that individuals treated for childhood cancer in 1940–2000 in France were recently hospitalised more than twice as often as the general population during a 13-year follow-up (2006–2018). This increase in the hospitalisation rate occurred among cancer survivors who had been treated with chemotherapy and/or radiotherapy. The hospitalisation rate was elevated for all ICD-10 groups of hospitalisation-related pathologies, although the RHR was the highest for hospitalisation related to neoplasms, endocrine conditions and circulatory system diseases.

Our results are consistent with similar studies performed in the USA, Canada, the Nordic countries and the Netherlands in which CCS experienced a higher hospitalisation rate compared with the general population of their countries^4–9^. In Europe, the RHR was generally higher: about two times higher in the Netherlands^6^ and the Nordic countries^9^, with a bed-day ratio of 5 in the Nordic countries, and an RHR of about 2.8 and a bed-day ratio of 3.7 in Scotland^17^, findings similar to our results. On the other hand, there was a lower hospitalisation rate in a small Utah cohort and in the large US Childhood Cancer Survivor Study (CCSS)^4,5^, probably because most of the children in those studies had been treated before the end of the 1970s. That time corresponds to the beginning of generalised use of combined chemotherapy^18^, which was more toxic than the previous single-agent chemotherapy.

Previous studies have shown that survivors are more at risk of hospitalisation due to neoplasms, recurrences and/or subsequent^4,7,9,17,19^. These results are similar to our findings in which both the AER and RHR were the highest in CCS. In two studies from North America, survivors were hospitalised more often because of blood disorders^4,19^. However, our results indicate that although blood disorders had a higher RHR, this category had a very low hospitalisation rate and AER. This outcome could be partially explained by the fact that our cohort did not include leukaemia survivors. One study from the UK reported that CCS had a four-fold risk of being hospitalised for cardiovascular disease compared to that expected from people of same age, sex and calendar year stratum^20^. Another study from the Netherlands^7^ showed a higher RHR but the lowest AER for endocrine conditions. These results are consistent with our findings but inconsistent with findings from the Nordic countries, where there was excessive hospitalisation mainly due to nervous system diseases^9^.

Researchers have reported a significantly higher hospitalisation rate in survivors of Hodgkin’s lymphoma^5^, CNS tumours^19^ and bone tumours^4,7^ compared with other primary cancer types. However, our results showed few variations in the RHR according to the primary cancer type, except for thyroid and CNS tumours. A high hospitalisation rate for nervous system diseases and congenital malformations have been also reported in CNS tumour survivors^17^, but the reclassification of neurofibromatosis from a tumour of uncertain behaviour in ICD-9 to congenital malformation in ICD-10 partially explains this excessive hospitalisation. On the contrary, in one study renal tumour survivors were not at additional risk of hospitalisation^4^, and in another one they had among the lowest hospitalisation rate^5^, which disagrees with our findings. However, this could be explained by the fact that we accounted for day hospital admissions, which include dialysis. In fact, our results show that their excessive hospitalisation comes from genitourinary system diseases.

Among FCCSS survivors, women experienced slightly higher hospitalisation and bed-day rates than men (respectively, 446.6 versus 363.5 per 1000 PY and 1242.3 versus 952.9 per 1000 PY, respectively). Compared with the reference population, women and men had a similar RHR and a higher AER for hospitalisation and bed-days, and women had a higher RBDR. These results are similar to a population-based cohort performed in Utah^4^, but not to another population-based cohort study performed in another US state, in which both the RHR and AER were higher for women^8^. Our findings are also different from the U.S. CCSS, in which women had a much lower RHR and AER than men^5^. In the Netherlands, two studies evidenced a higher hospitalisation rate but a lower RHR in women than in men^6,7^, whereas in the Scotland^17^ the standardised bed days ratio was almost the same in women and men.

Our findings are consistent with those of earlier studies in the Netherlands in which survivors initially treated with radiotherapy had a particularly increased hospitalisation rate for neoplasms, endocrine diseases and circulatory system diseases^7,21^. Another study in British Columbia, Canada, reported that hospital-related morbidity was elevated for all combinations of primary treatment and was highest for those who had received radiation, chemotherapy and surgery^19^. Our findings identified chemotherapy as a factor associated with hospitalisation especially for genitourinary system diseases, where cisplatin or ifosfamide have been established as treatment-related causes of chronic renal damage in CCS^22^.

Unexpectedly, we did not observe a variation in the RHR and RBDR according to the age at childhood cancer onset^21^. Our results about the variations in RHR and RBDR according to year of childhood cancer diagnosis and the age at the start of SNDS follow-up have to be interpreted with caution because these two variables are linked—that is, survivors treated in later years are likely to be older at the start of the follow-up. As a general matter, the differences in results among studies are hardly explained by variations in demographic and clinical characteristics. A more thorough investigation would require performing a meta-analysis. Our results should be interpreted with caution because the SNDS data are only available for 2006–2018, a period of time after the FCCSS recruitment period (1945–2000). Thus, a selection bias could occur in older patients at the time of the SNDS follow-up. For example, patients treated before 1970 who survived until 2006 are not representative of all patients treated before 1970 and correspond to a different distribution of the treatment types.

To our knowledge, this is the first detailed study of the hospitalisation of long-term CCS compared with the general population in France. We used a national administrative database, which provided comprehensive information on hospitalisations over 13 years in both CCS and their reference population. An advantage of our study is that we have accounted for hospitalisation in day hospital units. Admissions to day hospital units are mainly for chemotherapy, radiotherapy and extracorporeal dialysis. By considering in-patient bed-days, we could focus on more severe hospitalisation that required more medical care.

Our study is subject to some limitations. First, we were not able to identify hospitalisations related to relapse or metastasis of childhood cancer to the ones related to secondary neoplasms. Second, we considered only hospitalisation in conventional hospital units because information regarding rehabilitation and psychiatric institution hospitalisation was not available in the EGB sample, which constitutes our reference. Thus, we have underestimated the hospitalisation rate, especially mental-related hospitalisation. Nevertheless, the conventional hospital units treat more than 90% of all patients hospitalised in France^23^. Moreover, given that the EGB includes a population that does not receive health care and the data are stored for a period of 20 years^15^, the EGB allows researchers to carry out longitudinal studies of hospitalisations^24^. Third, we could not address the association between specific types of hospitalisations with specific modalities of therapy (e.g. chemotherapy and radiation doses) because this requires special considerations. We will perform these investigations in separate publications. Lastly, the FCCSS included only patients from five non-profit private cancer treatment centres in France, which are not representative of all French childhood cancer treatment centres. Nevertheless, we have found that this did not impact the long-term survivor's medical expenditure^25^.

In summary, we have shown that the hospitalisation and in-patient bed-day rates among CCS in France were more than twice higher than in the general population. The association of cancer treatment with the different types of hospitalisations suggests special attention should be paid to prevent long-term complications in all organ systems, especially among CCS treated with combined therapies.

## Supplementary Information

Supplementary Table 1.

Supplementary Table 2.

Supplementary Table 3.

Supplementary Table 4.

Supplementary Table 5.

Supplementary Table 6.

Supplementary Table 7.

Supplementary Table 8.

Supplementary Figure 1.

## Data Availability

The datasets generated and/or analysed during the current study are not publicly available because they contain potentially identifying patient information. However, the datasets are available from the corresponding author upon reasonable request.
