# Supplementary material for: Long-term hospitalisations in survivors of paediatric solid tumours in France
Source: Sci Rep. 2022 Oct 27;12:18068. doi: 10.1038/s41598-022-22689-w (PMC9613884; doi:10.1038/s41598-022-22689-w)
Supplement: Supplementary file 1 — Supplementary Table 1. [file 41598_2022_22689_MOESM1_ESM.docx]

| Supplementary Table 1. FCCSS patient characteristics according to the SNDS linkage status. | | |  |
| --- | --- | --- | --- |
|  | Linked to SNDS | Not Linked to SNDS | Chi2 |
|  | n (%) | n (%) |  |
| Total | 5583 * | 1235 * |  |
| Sex | 3050 (54.63) | 675 (54.66) | 0.9900 |
| Man | 2533 (45.37) | 560 (45.34) |  |
| Women |  |  |  |
| Age at January 2006 (Start date) |  |  |  |
| <20 | 1593 (28.53) | 309 (25.02) | <.0001 |
| 20-30 | 2115 (37.88) | 424 (34.33) |  |
| 31-40 | 1342 (24.04) | 314 (25.43) |  |
| >=41 | 533 (9.55) | 188 (15.22) |  |
| Status at December 2018 (Ending date ) | |  |  |
| Alive | 5184 (92.85) | 996 (80.65) | <.0001 |
| Death | 399 (7.15) | 239 (19.35) |  |
| Year of diagnosis |  |  |  |
| <1970 | 354 (6.34) | 112 (9.07) | <.0001 |
| 1970-1979 | 996 (17.84) | 275 (22.27) |  |
| 1980-1989 | 1858 (33.28) | 386 (31.26) |  |
| >=1990 | 2375 (42.54) | 462 (37.41) |  |
| Age at first cancer |  |  |  |
| 0-1 | 1323 (23.7) | 291 (23.56) | 0.5700 |
| 2-4 | 1308 (23.43) | 282 (22.83) |  |
| 5-9 | 1243 (22.26) | 260 (21.05) |  |
| 10-14 | 1133 (20.29) | 257 (20.81) |  |
| ≥15 | 576 (10.32) | 145 (11.74) |  |
| First primary cancer type |  |  |  |
| Other solid cancer | 324 (5.8) | 71 (5.75) | 0.5500 |
| Kidney tumors | 848 (15.19) | 195 (15.79) |  |
| Neuroblastoma | 775 (13.88) | 186 (15.06) |  |
| Lymphoma | 954 (17.09) | 212 (17.17) |  |
| Soft tissue sarcomas | 606 (10.85) | 124 (10.04) |  |
| Bone sarcomas | 489 (8.76) | 115 (9.31) |  |
| Central nervous system tumor | 717 (12.84) | 142 (11.5) |  |
| Gonadal/Germ cell tumours | 343 (6.14) | 69 (5.59) |  |
| Thyroid tumor | 49 (0.88) | 12 (0.97) |  |
| Retinoblastoma | 478 (8.56) | 108 (8.74) |  |
| Treatment Received |  |  |  |
| No radiotherapy or chemotherapy | 731 (13.09) | 142 (11.5) | 0.0100 |
| Radiotherapy | 711 (12.74) | 182 (14.74) |  |
| Chemotherapy | 2062 (36.93) | 409 (33.12) |  |
| Radiotherapy and Chemotherapy | 2079 (37.24) | 502 (40.65) |  |

* Only 6818 patients were eligible, i.e. alive in 2006 at the start of the SNDS.
