# Supplementary material for: Long-term hospitalisations in survivors of paediatric solid tumours in France
Source: Sci Rep. 2022 Oct 27;12:18068. doi: 10.1038/s41598-022-22689-w (PMC9613884; doi:10.1038/s41598-022-22689-w)
Supplement: Supplementary file 2 — Supplementary Table 2. [file 41598_2022_22689_MOESM2_ESM.docx]

| Supplementary Table 2. Main primary diagnosis of childhood cancer survivors by chapters of ICD-10. |  |  |
| --- | --- | --- |
| Chapter I Certain infectious and parasitic diseases | n | % |
| Infectious gastroenteritis and colitis, unspecified | 41 | 16.73 |
| Erysipelas | 31 | 12.65 |
| Anogenital (venereal) warts | 26 | 10.61 |
| Chronic viral hepatitis B without delta-agent | 12 | 4.90 |
| Sepsis due to Staphylococcus aureus | 9 | 3.67 |
| Sepsis, unspecified organism | 9 | 3.67 |
| Sepsis due to other Gram-negative organisms | 8 | 3.27 |
| Chronic viral hepatitis C | 7 | 2.86 |
| Other and unspecified infectious diseases | 7 | 2.86 |
| Others | 95 | 38.78 |
| Chapter II Neoplasms |  |  |
| Encounter for antineoplastic chemotherapy and immunotherapy | 3269 | 32.37 |
| Encounter for antineoplastic radiation therapy | 2595 | 25.69 |
| Personal history of malig neoplasms of organs and systems | 672 | 6.65 |
| Encounter for other aftercare | 609 | 6.03 |
| Encounter for follow-up examination after completed treatment for malignant neoplasm | 237 | 2.35 |
| Personal history of other malignant neoplasms of lymphoid, hematopoietic and related tissues | 187 | 1.85 |
| Personal history of malignant neoplasm of urinary tract | 144 | 1.43 |
| Malignant neoplasm of thyroid gland | 108 | 1.07 |
| Benign neoplasm of thyroid gland | 97 | 0.96 |
| Others | 2182 | 21.60 |
| Chapter III Diseases of the blood and blood-forming organs |  |  |
| Drug-induced aplastic anemia | 41 | 17.30 |
| Anemia in other chronic diseases classified elsewhere | 20 | 8.44 |
| Iron deficiency anemia secondary to blood loss (chronic) | 15 | 6.33 |
| Anemia in neoplastic disease | 13 | 5.49 |
| Iron deficiency anemia, unspecified | 12 | 5.06 |
| Other specified anemias | 12 | 5.06 |
| Acute posthemorrhagic anemia | 10 | 4.22 |
| Immunodeficiency, unspecified | 10 | 4.22 |
| Secondary thrombocytopenia | 10 | 4.22 |
| Others | 94 | 39.66 |
| Chapter IV Endocrine, nutritional and metabolic diseases |  |  |
| Hypopituitarism | 153 | 18.43 |
| Postprocedural hypopituitarism | 58 | 6.99 |
| Nontoxic multinodular goiter | 56 | 6.75 |
| Type 1 diabetes mellitus without complications | 52 | 6.27 |
| Type 2 diabetes mellitus without complications | 51 | 6.14 |
| Nontoxic single thyroid nodule | 40 | 4.82 |
| Obesity due to excess calories | 29 | 3.49 |
| Morbid (severe) obesity due to excess calories | 28 | 3.37 |
| Drug-induced hypopituitarism | 24 | 2.89 |
| Others | 339 | 40.84 |
| Chapter V Mental and behavioural disorders |  |  |
| Alcohol related disorders | 123 | 41.00 |
| Adjustment disorders | 16 | 5.33 |
| Alcohol dependence | 11 | 3.67 |
| Major depressv disord, single epsd, sev w/o psych features | 9 | 3.00 |
| Anorexia nervosa | 8 | 2.67 |
| Other mental disorders due to known physiological condition | 8 | 2.67 |
| Generalized anxiety disorder | 7 | 2.33 |
| Major depressive disorder, single episode, unspecified | 7 | 2.33 |
| Other anxiety disorders | 6 | 2.00 |
| Others | 105 | 35.00 |
| Chapter VI Diseases of the nervous system |  |  |
| Carpal tunnel syndrome | 70 | 8.72 |
| Local-rel (focal) symptc epilepsy w complex partial seizure | 57 | 7.10 |
| Epilepsy, unspecified | 52 | 6.48 |
| Spastic hemiplegia | 41 | 5.11 |
| Generalized idiopathic epilepsy and epileptic syndromes | 31 | 3.86 |
| Nerve root and plexus compressions in diseases classd elswh | 27 | 3.36 |
| Paraplegia (paraparesis) and quadriplegia (quadriparesis) | 24 | 2.99 |
| Cluster headaches and oth trigeminal autonm cephalgias (TAC | 19 | 2.37 |
| Multiple sclerosis | 18 | 2.24 |
| Others | 464 | 57.78 |
| Chapter VII Diseases of the eye and adnexa |  |  |
| Infantile and juvenile cataract | 35 | 10.74 |
| Enophthalmos | 30 | 9.20 |
| Ptosis of eyelid | 22 | 6.75 |
| Other disorders of globe | 21 | 6.44 |
| Unspecified cataract | 16 | 4.91 |
| Other specified cataract | 13 | 3.99 |
| Exotropia | 10 | 3.07 |
| Other disorders of orbit | 10 | 3.07 |
| Esotropia | 8 | 2.45 |
| Others | 161 | 49.39 |
| Chapter VIII Diseases of the ear and mastoid process |  |  |
| Cholesteatoma of middle ear | 13 | 11.93 |
| Chronic serous otitis media | 12 | 11.01 |
| Sensorineural hearing loss, bilateral | 9 | 8.26 |
| Other peripheral vertigo | 8 | 7.34 |
| Sudden idiopathic hearing loss | 8 | 7.34 |
| Unspecified sensorineural hearing loss | 7 | 6.42 |
| Other chronic nonsuppurative otitis media | 4 | 3.67 |
| Other perforations of tympanic membrane | 4 | 3.67 |
| Benign paroxysmal vertigo | 3 | 2.75 |
| Others | 41 | 37.61 |
| Chapter IX Diseases of the circulatory system |  |  |
| Heart transplant status | 345 | 18.16 |
| Encounter for adjustment and management of VAD | 213 | 11.21 |
| Heart failure | 125 | 6.58 |
| Dilated cardiomyopathy | 71 | 3.74 |
| Asymptomatic varicose veins of lower extremities | 68 | 3.58 |
| Left ventricular failure | 57 | 3.00 |
| Atherosclerotic heart disease of native coronary artery | 37 | 1.95 |
| Unstable angina | 36 | 1.89 |
| Cerebral infarction, unspecified | 31 | 1.63 |
| Others | 917 | 48.26 |
| Chapter X Diseases of the respiratory system |  |  |
| Acute respiratory failure | 47 | 7.82 |
| Chronic respiratory failure | 45 | 7.49 |
| Pneumonitis due to inhalation of food and vomit | 38 | 6.32 |
| Unspecified bacterial pneumonia | 26 | 4.33 |
| Deviated nasal septum | 21 | 3.49 |
| Pneumonia, unspecified organism | 21 | 3.49 |
| Pleural effusion, not elsewhere classified | 20 | 3.33 |
| Acute bronchitis, unspecified | 18 | 3.00 |
| Bronchopneumonia, unspecified organism | 15 | 2.50 |
| Others | 350 | 58.24 |
| Chapter XI Diseases of the digestive system |  |  |
| Embedded teeth | 159 | 7.52 |
| Polyp of colon | 87 | 4.12 |
| Other and unspecified intestinal obstruction | 73 | 3.45 |
| Other gastritis | 62 | 2.93 |
| Diverticular disease of lg int w/o perforation or abscess | 54 | 2.56 |
| Dental caries | 44 | 2.08 |
| Calculus of gallbladder without cholecystitis | 42 | 1.99 |
| Intestinal adhesions w obst (postprocedural) (postinfection | 42 | 1.99 |
| Unilateral inguinal hernia, without obstruction or gangrene | 41 | 1.94 |
| Others | 1509 | 71.42 |
| Chapter XII Diseases of the skin and subcutaneous tissue |  |  |
| Pilonidal cyst and sinus with abscess | 39 | 9.92 |
| Scar conditions and fibrosis of skin | 34 | 8.65 |
| Cutaneous abscess, furuncle and carbuncle of limb | 32 | 8.14 |
| Cutaneous abscess, furuncle and carbuncle of trunk | 32 | 8.14 |
| Cellulitis and acute lymphangitis of finger and toe | 30 | 7.63 |
| Ingrowing nail | 27 | 6.87 |
| Pilonidal cyst and sinus without abscess | 22 | 5.60 |
| Epidermal cyst | 21 | 5.34 |
| Oth local infections of the skin and subcutaneous tissue | 18 | 4.58 |
| Others | 138 | 35.11 |
| Chapter XIII Diseases of the musculoskeletal system and connective tissue |  |  |
| Low back pain | 47 | 5.33 |
| Thor, thrclm & lumbosacr intvrt disc disord w radiculopathy | 35 | 3.97 |
| Hallux valgus (acquired) | 29 | 3.29 |
| Disorder of continuity of bone | 25 | 2.84 |
| Other dorsalgia | 20 | 2.27 |
| Other specified disorders of muscle | 19 | 2.16 |
| Unilateral primary osteoarthritis of hip | 17 | 1.93 |
| Chronic instability of knee, right knee | 16 | 1.82 |
| Radiculopathy, lumbar region | 15 | 1.70 |
| Others | 658 | 74.69 |
| Chapter XIV Diseases of the genitourinary system |  |  |
| Encounter for care involving renal dialysis | 1848 | 53.38 |
| Kidney transplant status | 151 | 4.36 |
| Acute tubulo-interstitial nephritis | 107 | 3.09 |
| Calculus of kidney | 90 | 2.60 |
| Calculus of ureter | 73 | 2.11 |
| Unspecified renal colic | 65 | 1.88 |
| Chronic kidney disease (CKD) | 60 | 1.73 |
| Polyp of corpus uteri | 53 | 1.53 |
| Other and unspecified ovarian cysts | 41 | 1.18 |
| Others | 974 | 28.13 |
| Chapter XVII Certain conditions originating in the perinatal period |  |  |
| Neurofibromatosis (nonmalignant) | 70 | 45.75 |
| Prominent ear | 6 | 3.92 |
| Congenital malformations of other endocrine glands | 4 | 2.61 |
| Other atresia and stenosis of urethra and bladder neck | 4 | 2.61 |
| Arteriovenous malformation (peripheral) | 3 | 1.96 |
| Arteriovenous malformation of cerebral vessels | 3 | 1.96 |
| Congenital facial asymmetry | 3 | 1.96 |
| Other congenital malformations of breast | 3 | 1.96 |
| Other phakomatoses, not elsewhere classified | 3 | 1.96 |
| Others | 54 | 35.29 |
| Chapter XVIII Symptoms, signs and abnormal clinical and laboratory findings, not elsewhere classified |  |  |
| Abdominal and pelvic pain | 140 | 12.07 |
| Pain, unspecified | 93 | 8.02 |
| Pain in throat and chest | 74 | 6.38 |
| Malaise and fatigue | 68 | 5.86 |
| Pain localized to upper abdomen | 64 | 5.52 |
| Ascites | 53 | 4.57 |
| Headache | 44 | 3.79 |
| Fever, unspecified | 43 | 3.71 |
| Dyspnea | 32 | 2.76 |
| Others | 549 | 47.33 |
| Chapter XIX Injury, poisoning and certain other consequences of external causes |  |  |
| Mechanical complication of internal joint prosthesis | 66 | 6.58 |
| Benzodiazepines | 52 | 5.18 |
| Concussion | 32 | 3.19 |
| Open wound of thumb without damage to nail | 25 | 2.49 |
| Unspecified fracture of the lower end of radius | 23 | 2.29 |
| Traumatic subdural hemorrhage | 22 | 2.19 |
| Mechanical comp of cardiac and vascular devices and implant | 21 | 2.09 |
| Sprain of cruciate ligament of knee | 21 | 2.09 |
| Inj extensor musc/fasc/tend and unsp finger at wrs/hnd lv | 19 | 1.89 |
| Others | 722 | 71.98 |
| Chapter XXI Factors influencing health status and contact with health services |  |  |
| Encounter for follow-up examination after completed treatment for conditions other than malignant neoplasm | 728 | 24.41 |
| Encounter for other aftercare | 330 | 11.07 |
| Encounter for examination and observation for oth reasons | 280 | 9.39 |
| Encounter for other specified aftercare | 279 | 9.36 |
| Encounter for palliative care | 164 | 5.50 |
| Encntr for plast/recnst surg fol med proc or healed injury | 142 | 4.76 |
| Orthopedic aftercare | 105 | 3.52 |
| Encounter for adjustment and management of implanted device | 103 | 3.45 |
| Encounter for breast reconstruction following mastectomy | 66 | 2.21 |
| Others | 785 | 26.32 |
