# Supplementary material for: Long-term hospitalisations in survivors of paediatric solid tumours in France
Source: Sci Rep. 2022 Oct 27;12:18068. doi: 10.1038/s41598-022-22689-w (PMC9613884; doi:10.1038/s41598-022-22689-w)
Supplement: Supplementary file 3 — Supplementary Table 3. [file 41598_2022_22689_MOESM3_ESM.docx]

| Supplementary Table 3. Relative hospitalisation and bed-days ratios by type of hospitalization and type of primary cancer. | | | | | | |  |  |  |  |
| --- | --- | --- | --- | --- | --- | --- | --- | --- | --- | --- |
|  | Hospitalizations | | | | | | | | | |
|  | Other solid cancer | Kidney tumors | Neuroblastoma | Lymphoma | Soft tissue sarcomas | Bone sarcomas | Central nervous system tumor | Gonadal/Germ cell tumours | Thyroid tumor | Retinoblastoma |
| Total | 2.71 (2.58-2.84) | 2.63 (2.56-2.71) | 2.58 (2.49-2.67) | 2.36 (2.3-2.43) | 2.23 (2.15-2.31) | 2.14 (2.06-2.23) | 3.56 (3.45-3.67) | 1.85 (1.75-1.94) | 0.81 (0.69-0.95) | 2.72 (2.59-2.85) |
| Infections | 2.53 (1.38-4.24) | 4 (3.07-5.11) | 2.05 (1.36-2.97) | 2.34 (1.69-3.15) | 2 (1.28-2.98) | 2.5 (1.6-3.72) | 2.34 (1.58-3.34) | 1.49 (0.72-2.74) | 1.98 (0.24-7.16) | 0.79 (0.32-1.64) |
| Neoplasms | 3.85 (3.54-4.19) | 2.68 (2.54-2.83) | 3.36 (3.13-3.61) | 3.85 (3.69-4.02) | 2.96 (2.79-3.14) | 3.73 (3.52-3.94) | 5.15 (4.9-5.41) | 2.18 (1.99-2.38) | 0.55 (0.38-0.77) | 10.32 (9.65-11.03) |
| Haematological | 4.23 (2.37-6.97) | 4.35 (3.2-5.76) | 3.42 (2.36-4.81) | 5.16 (3.96-6.61) | 1.24 (0.62-2.22) | 2.77 (1.67-4.33) | 3.02 (1.99-4.39) | 1.26 (0.46-2.75) | 0 (.-2.59) | 3.48 (1.99-5.65) |
| Endocrine | 2.1 (1.42-3) | 2.78 (2.27-3.37) | 2.61 (2.06-3.27) | 2.95 (2.46-3.5) | 2.27 (1.74-2.91) | 1.55 (1.08-2.15) | 10.45 (9.29-11.7) | 3.78 (2.91-4.81) | 1.47 (0.4-3.75) | 2.02 (1.33-2.94) |
| Mental | 2.39 (1.59-3.45) | 1.2 (0.89-1.57) | 0.79 (0.53-1.14) | 0.94 (0.68-1.26) | 1.81 (1.35-2.36) | 0.38 (0.18-0.71) | 1.7 (1.28-2.23) | 0.65 (0.33-1.13) | 4.45 (1.92-8.76) | 0.77 (0.42-1.3) |
| Neurological | 2.79 (2.06-3.7) | 1.09 (0.83-1.41) | 2.56 (2.06-3.15) | 1.38 (1.11-1.7) | 1.11 (0.81-1.49) | 0.93 (0.64-1.32) | 10.48 (9.44-11.61) | 1.6 (1.11-2.22) | 2.18 (1.05-4.01) | 1.72 (1.12-2.51) |
| Ocular | 0.83 (0.36-1.63) | 0.77 (0.49-1.16) | 1.3 (0.76-2.09) | 0.81 (0.55-1.15) | 1.4 (0.98-1.94) | 0.43 (0.21-0.8) | 3 (2.29-3.87) | 0.51 (0.23-0.97) | 0.7 (0.23-1.65) | 23.41 (19.52-27.86) |
| Auditory | 4.62 (2.64-7.51) | 0.9 (0.39-1.78) | 0.13 (0-0.72) | 1.51 (0.84-2.48) | 1.7 (0.85-3.04) | 1.2 (0.44-2.61) | 6.4 (4.63-8.62) | 1.6 (0.59-3.48) | 1.34 (0.03-7.47) | 0.32 (0.04-1.14) |
| Cardiovascular | 2.75 (2.21-3.38) | 3.06 (2.73-3.43) | 4.33 (3.75-4.97) | 3.6 (3.28-3.96) | 4.86 (4.38-5.39) | 2.46 (2.09-2.86) | 2.69 (2.31-3.13) | 2.32 (1.88-2.83) | 0.44 (0.14-1.03) | 3.89 (3-4.97) |
| Pulmonary | 2.3 (1.61-3.19) | 2.58 (2.12-3.11) | 1.01 (0.72-1.38) | 2.62 (2.19-3.1) | 1.48 (1.1-1.96) | 1.13 (0.77-1.6) | 4.89 (4.17-5.7) | 0.75 (0.41-1.26) | 1.21 (0.33-3.09) | 0.83 (0.5-1.3) |
| Gastrointestinal | 2 (1.72-2.32) | 1.58 (1.43-1.75) | 1.05 (0.91-1.2) | 1.44 (1.3-1.58) | 1.3 (1.14-1.48) | 1.08 (0.92-1.26) | 1.32 (1.15-1.5) | 1.49 (1.27-1.74) | 0.93 (0.56-1.45) | 0.86 (0.72-1.04) |
| Skin | 2.71 (1.8-3.91) | 1.32 (0.93-1.81) | 1.84 (1.39-2.41) | 2.2 (1.73-2.76) | 2.4 (1.81-3.14) | 2.61 (1.89-3.52) | 1.85 (1.34-2.49) | 3.23 (2.29-4.43) | 0.62 (0.02-3.43) | 1.03 (0.63-1.59) |
| Musculoskeletal | 0.97 (0.7-1.31) | 1.21 (1.02-1.42) | 1.87 (1.59-2.2) | 0.97 (0.82-1.15) | 1.04 (0.85-1.27) | 1.41 (1.17-1.7) | 1.13 (0.92-1.38) | 0.87 (0.64-1.16) | 1.36 (0.78-2.21) | 0.83 (0.56-1.19) |
| Genitourinary | 3.36 (2.89-3.89) | 6.66 (6.29-7.05) | 7.44 (6.96-7.95) | 1.31 (1.16-1.46) | 2.12 (1.89-2.36) | 1.01 (0.83-1.23) | 1.23 (1.02-1.48) | 3.08 (2.68-3.52) | 0.34 (0.15-0.64) | 1.96 (1.64-2.32) |
| Congenital Malformations | 0.96 (0.12-3.47) | 2.39 (1.37-3.89) | 1.43 (0.76-2.45) | 1.88 (0.94-3.36) | 2.57 (1.33-4.49) | 1.29 (0.35-3.3) | 14.34 (11.22-18.06) | 5.36 (2.86-9.17) | 4.95 (0.6-17.89) | 0.92 (0.4-1.81) |
| Symptoms Unclassified | 2.53 (2-3.15) | 2.48 (2.17-2.81) | 1.57 (1.29-1.89) | 1.99 (1.72-2.28) | 1.73 (1.43-2.06) | 1.39 (1.1-1.74) | 3.62 (3.18-4.12) | 1.02 (0.73-1.38) | 1.38 (0.66-2.53) | 1.08 (0.77-1.46) |
| Injury - Poisoning | 1.3 (0.97-1.72) | 1.14 (0.94-1.36) | 1.13 (0.94-1.36) | 1.44 (1.24-1.66) | 1.33 (1.1-1.6) | 2.36 (2.01-2.75) | 1.77 (1.5-2.07) | 1.12 (0.83-1.48) | 1.16 (0.5-2.28) | 0.69 (0.5-0.91) |
| Other Factors | 3.02 (2.63-3.45) | 2.72 (2.49-2.97) | 2.15 (1.91-2.41) | 2.32 (2.12-2.53) | 2.55 (2.29-2.83) | 1.97 (1.73-2.24) | 4.49 (4.13-4.87) | 1.77 (1.49-2.08) | 0.89 (0.46-1.56) | 2.4 (2.04-2.81) |

| Continued Supplementary Table 3. Relative hospitalisation and bed-days ratios by type of hospitalization and type of primary cancer. | | | | | | |  |  |  |  |
| --- | --- | --- | --- | --- | --- | --- | --- | --- | --- | --- |
|  |  | Bed-days | | | | | | | | |
|  | Other solid cancer | Kidney tumors | Neuroblastoma | Lymphoma | Soft tissue sarcomas | Bone sarcomas | Central nervous system tumor | Gonadal/Germ cell tumours | Thyroid tumor | Retinoblastoma |
| Total | 3.25 (3.14-3.35) | 3.5 (3.44-3.56) | 3.2 (3.12-3.27) | 2.9 (2.85-2.95) | 2.82 (2.76-2.88) | 2.71 (2.65-2.78) | 6.29 (6.2-6.39) | 3.13 (3.04-3.22) | 1.17 (1.06-1.29) | 5.17 (5.03-5.31) |
| Infections | 2.32 (1.75-3.03) | 7.66 (7.06-8.3) | 1.11 (0.84-1.43) | 2.07 (1.81-2.36) | 2.02 (1.69-2.4) | 5.61 (4.91-6.37) | 2.46 (2.05-2.93) | 1.58 (1.2-2.04) | 2.73 (1.49-4.58) | 0.53 (0.29-0.89) |
| Neoplasms | 4.47 (4.18-4.76) | 3.02 (2.9-3.15) | 3.1 (2.9-3.3) | 4.33 (4.18-4.48) | 2.84 (2.7-2.99) | 3.39 (3.22-3.57) | 6.92 (6.68-7.15) | 3.1 (2.9-3.3) | 1.52 (1.27-1.81) | 12.2 (11.55-12.88) |
| Haematological | 14.84 (12.47-17.53) | 3.26 (2.72-3.87) | 2.58 (2.1-3.15) | 3.71 (3.19-4.3) | 1 (0.68-1.43) | 2.2 (1.65-2.87) | 3.37 (2.76-4.07) | 3.77 (2.95-4.75) | 0 (.-0.66) | 2.95 (2.19-3.89) |
| Endocrine | 1.38 (1.09-1.72) | 2.26 (2.02-2.51) | 2.02 (1.76-2.31) | 1.62 (1.44-1.81) | 2.27 (2-2.58) | 1.05 (0.85-1.29) | 6.09 (5.63-6.57) | 2.46 (2.1-2.87) | 1.19 (0.65-1.99) | 1.62 (1.26-2.04) |
| Mental | 0.78 (0.49-1.18) | 1.37 (1.16-1.62) | 1.01 (0.82-1.24) | 1.55 (1.34-1.78) | 0.83 (0.64-1.06) | 0.18 (0.09-0.31) | 2.25 (1.94-2.59) | 0.54 (0.34-0.82) | 2.59 (1.34-4.52) | 0.5 (0.32-0.75) |
| Neurological | 4.51 (3.87-5.24) | 1.66 (1.44-1.91) | 2.92 (2.57-3.31) | 2.54 (2.3-2.8) | 1.41 (1.19-1.66) | 0.97 (0.77-1.21) | 32.19 (31.02-33.39) | 2.37 (1.96-2.84) | 3.09 (2.15-4.3) | 4.88 (4.16-5.69) |
| Ocular | 0.33 (0.04-1.21) | 2.73 (2-3.63) | 2.05 (1.25-3.16) | 1.96 (1.43-2.63) | 5.65 (4.47-7.04) | 0 (.-0.28) | 7.57 (6.11-9.27) | 1.77 (1.03-2.84) | 0 (.-1.53) | 25.04 (21.19-29.38) |
| Auditory | 3.99 (2.63-5.81) | 0.85 (0.47-1.43) | 0 (.-0.38) | 1.93 (1.34-2.68) | 1.93 (1.16-3.02) | 0.79 (0.32-1.62) | 12.34 (10.19-14.82) | 1.49 (0.74-2.67) | 7.12 (3.07-14.03) | 0 (.-0.54) |
| Cardiovascular | 4.55 (4.14-4.99) | 3.64 (3.43-3.85) | 8.4 (7.91-8.91) | 5.68 (5.46-5.91) | 5.5 (5.22-5.81) | 2.31 (2.12-2.52) | 6.45 (6.12-6.8) | 2.46 (2.2-2.73) | 0.57 (0.35-0.87) | 3.24 (2.76-3.79) |
| Pulmonary | 4.33 (3.85-4.86) | 3.73 (3.45-4.03) | 1.98 (1.73-2.26) | 3.64 (3.4-3.89) | 1.52 (1.33-1.73) | 4.11 (3.77-4.49) | 9 (8.5-9.51) | 0.45 (0.32-0.62) | 0.55 (0.26-1.01) | 1.4 (1.12-1.73) |
| Gastrointestinal | 3.03 (2.74-3.34) | 3.62 (3.43-3.81) | 1.76 (1.6-1.94) | 2.11 (1.98-2.25) | 3.42 (3.21-3.65) | 1.23 (1.09-1.38) | 2.14 (1.98-2.32) | 3.42 (3.15-3.71) | 1.33 (0.98-1.75) | 1.63 (1.42-1.86) |
| Skin | 1.44 (0.92-2.17) | 1.85 (1.5-2.26) | 2.13 (1.73-2.6) | 2.57 (2.2-2.99) | 3.02 (2.53-3.59) | 3.57 (2.95-4.27) | 3.19 (2.67-3.78) | 2.14 (1.66-2.71) | 0 (.-1.06) | 1.89 (1.33-2.6) |
| Musculoskeletal | 0.63 (0.49-0.8) | 1.62 (1.48-1.78) | 2.7 (2.47-2.95) | 1.24 (1.13-1.36) | 1.36 (1.22-1.52) | 4.23 (3.96-4.52) | 1.3 (1.15-1.46) | 0.88 (0.73-1.05) | 2.3 (1.84-2.83) | 1.19 (0.96-1.46) |
| Genitourinary | 2.03 (1.76-2.33) | 9.85 (9.51-10.19) | 9.63 (9.18-10.1) | 2.38 (2.23-2.52) | 3.41 (3.19-3.63) | 0.74 (0.63-0.86) | 2.32 (2.12-2.53) | 11.67 (11.08-12.29) | 0.21 (0.1-0.38) | 19.39 (18.54-20.27) |
| Congenital Malformations | 0.76 (0.31-1.58) | 3.51 (2.63-4.57) | 2.31 (1.67-3.13) | 1.64 (1.02-2.51) | 1.2 (0.64-2.05) | 1.05 (0.39-2.29) | 33.88 (30.27-37.81) | 2.42 (1.21-4.33) | 2.07 (0.05-11.54) | 1.55 (1-2.31) |
| Symptoms Unclassified | 3.88 (3.31-4.52) | 4.07 (3.74-4.43) | 2.16 (1.86-2.48) | 3.07 (2.8-3.35) | 3.82 (3.46-4.2) | 2.64 (2.31-3) | 8.17 (7.6-8.77) | 3.41 (2.93-3.94) | 0.99 (0.53-1.69) | 1.95 (1.59-2.37) |
| Injury - Poisoning | 2.47 (2.16-2.81) | 1.11 (0.99-1.25) | 1.57 (1.41-1.74) | 1.54 (1.42-1.67) | 1.94 (1.77-2.13) | 5.39 (5.06-5.74) | 5.7 (5.38-6.03) | 1.27 (1.08-1.49) | 0.33 (0.13-0.68) | 0.59 (0.47-0.72) |
| Other Factors | 4.85 (4.38-5.36) | 2.23 (2.06-2.42) | 2.23 (2.01-2.47) | 2.29 (2.13-2.46) | 3.31 (3.06-3.59) | 2.44 (2.21-2.69) | 8.88 (8.44-9.33) | 3.5 (3.16-3.87) | 0.7 (0.39-1.16) | 10.31 (9.51-11.16) |
