# Supplementary material for: Long-term hospitalisations in survivors of paediatric solid tumours in France
Source: Sci Rep. 2022 Oct 27;12:18068. doi: 10.1038/s41598-022-22689-w (PMC9613884; doi:10.1038/s41598-022-22689-w)
Supplement: Supplementary file 4 — Supplementary Table 4. [file 41598_2022_22689_MOESM4_ESM.docx]

| Supplementary Table 4. Relative hospitalisation rates and AER by attained age in 2006 groups. | | | | |  |  |  |  |
| --- | --- | --- | --- | --- | --- | --- | --- | --- |
|  | attained age <20 | | attained age 20-30 | | attained age 31-40 | | attained age >=41 | |
|  | RHR | AER | RHR | AER | RHR | AER | RHR | AER |
| Sex |  |  |  |  |  |  |  |  |
| Man | 2.26 (2.16-2.37) | 105.6 | 3.81 (3.71-3.91) | 273.8 | 3.14 (3.04-3.23) | 295.7 | 2.54 (2.44-2.65) | 414.4 |
| Women | 2.44 (2.34-2.55) | 127.5 | 3.62 (3.52-3.72) | 328.9 | 2.95 (2.86-3.04) | 378.9 | 2.74 (2.63-2.84) | 528.6 |
| Status at December 2018 (Ending date ) |  |  |  |  |  |  |  |  |
| Alive | 1.8 (1.74-1.87) | 69.5 | 2.67 (2.61-2.73) | 191.1 | 2.24 (2.18-2.29) | 212.6 | 1.71 (1.64-1.77) | 224.6 |
| Death | 43.62 (40.92-46.46) | 2988.6 | 53.44 (51.53-55.41) | 3207.4 | 20.12 (19.29-20.97) | 2068.5 | 11 (10.5-11.51) | 1787.1 |
| Year of diagnosis |  |  |  |  |  |  |  |  |
| <1970 | . | . | . | . | 2.16 (1.95-2.38) | 238.9 | 2.41 (2.31-2.5) | 440.3 |
| 1970-1979 | . | . | 4.7 (4.44-4.97) | 494.6 | 3.3 (3.2-3.4) | 376.8 | 3.15 (3.02-3.28) | 533.8 |
| 1980-1989 | 4.39 (4.03-4.78) | 286.9 | 3.73 (3.63-3.83) | 303.6 | 2.98 (2.88-3.08) | 319.3 | 2.12 (1.66-2.68) | 214.7 |
| >=1990 | 2.19 (2.12-2.27) | 102.6 | 3.33 (3.22-3.44) | 248.2 | 1.9 (1.66-2.17) | 133.7 | . | . |
| Age at first cancer |  |  |  |  |  |  |  |  |
| 0-1 | 2.15 (2.04-2.26) | 97.6 | 4.14 (3.97-4.31) | 368.3 | 2.8 (2.65-2.97) | 323.9 | 2.74 (2.51-2.98) | 473.7 |
| 2-4 | 2.08 (1.96-2.20) | 94.4 | 3.74 (3.6-3.89) | 304.8 | 3.04 (2.88-3.21) | 328.4 | 2.7 (2.52-2.89) | 528.8 |
| 5-9 | 2.81 (2.62-2.99) | 154.4 | 4.17 (4.01-4.34) | 334.9 | 3.48 (3.34-3.63) | 396.7 | 2.29 (2.15-2.44) | 358.0 |
| 10-14 | 3.61 (3.26-3.98) | 225.4 | 3.27 (3.13-3.41) | 254.4 | 2.99 (2.87-3.12) | 332.8 | 3.26 (3.12-3.4) | 628.8 |
| ≥15 | . | . | 2.64 (2.47-2.81) | 180.5 | 2.48 (2.34-2.62) | 243.3 | 1.62 (1.47-1.78) | 190.9 |
| First primary cancer type |  |  |  |  |  |  |  |  |
| Other solid cancer | 2.35 (2.03-2.72) | 113.7 | 3.8 (3.52-4.1) | 281.7 | 2.89 (2.62-3.17) | 326.0 | 3.79 (3.39-4.23) | 725.4 |
| Kidney tumors | 1.87 (1.7-2.05) | 72.5 | 4.05 (3.86-4.25) | 362.1 | 2.98 (2.82-3.14) | 339.5 | 3.61 (3.4-3.82) | 705.9 |
| Neuroblastoma | 1.36 (1.24-1.49) | 30.6 | 4.17 (3.97-4.37) | 368.6 | 2.37 (2.19-2.56) | 238.1 | 3.2 (2.86-3.58) | 565.2 |
| Lymphoma | 2.1 (1.87-2.36) | 95.7 | 2.93 (2.79-3.08) | 205.7 | 2.92 (2.79-3.06) | 331.1 | 3.32 (3.14-3.51) | 648.6 |
| Soft tissue sarcomas | 2.8 (2.53-3.08) | 157.1 | 3.92 (3.7-4.16) | 329.4 | 2.7 (2.54-2.86) | 278.0 | 1.67 (1.52-1.83) | 216.7 |
| Bone sarcomas | 3.52 (3.08-4.01) | 202.2 | 3.33 (3.13-3.54) | 259.6 | 2.39 (2.21-2.58) | 222.3 | 2.15 (1.97-2.34) | 347.2 |
| Central nervous system tumor | 4.09 (3.83-4.36) | 266.1 | 4.72 (4.5-4.96) | 381.9 | 4.99 (4.72-5.28) | 560.6 | 2.72 (2.48-2.97) | 417.3 |
| Gonadal/Germ cell tumours | 3.05 (2.58-3.58) | 149.8 | 2.02 (1.84-2.21) | 120.5 | 3.01 (2.77-3.25) | 334.4 | 1.49 (1.3-1.7) | 157.6 |
| Thyroid tumor | 0.97 (0.36-2.11) | -2.4 | 1.89 (1.43-2.45) | 102.6 | 1.56 (1.01-2.3) | 114.6 | 0.86 (0.66-1.1) | -53.5 |
| Retinoblastoma | 2.21 (2.07-2.37) | 108.6 | 4.23 (3.92-4.56) | 377.9 | 4.02 (3.43-4.69) | 483.3 | 0.61 (0.33-1.02) | -140.5 |
| Treatment Received |  |  |  |  |  |  |  |  |
| No radiotherapy or chemotherapy | 1.52 (1.39-1.66) | 45.0 | 1.86 (1.73-2) | 97.9 | 1.41 (1.27-1.56) | 71.2 | 1.01 (0.88-1.17) | 4.5 |
| Radiotherapy | 4.05 (3.69-4.43) | 279.2 | 3.96 (3.74-4.19) | 309.5 | 4.38 (4.15-4.61) | 537.8 | 2.22 (2.11-2.33) | 372.0 |
| Chemotherapy | 1.8 (1.71-1.89) | 67.7 | 2.95 (2.85-3.04) | 222.2 | 2.37 (2.26-2.49) | 214.5 | 2.2 (2-2.42) | 371.8 |
| Radiotherapy and Chemotherapy | 3.44 (3.27-3.61) | 209.1 | 5.18 (5.04-5.32) | 456.0 | 3.29 (3.2-3.39) | 393.6 | 3.77 (3.62-3.91) | 677.2 |
