# Supplementary material for: Long-term hospitalisations in survivors of paediatric solid tumours in France
Source: Sci Rep. 2022 Oct 27;12:18068. doi: 10.1038/s41598-022-22689-w (PMC9613884; doi:10.1038/s41598-022-22689-w)
Supplement: Supplementary file 5 — Supplementary Table 5. [file 41598_2022_22689_MOESM5_ESM.docx]

| Supplementary Table 5. Relative Bed-days ratio and AER by attained age in 2006 groups. | | | |  |  |  |  |  |
| --- | --- | --- | --- | --- | --- | --- | --- | --- |
|  | attained age <20 | | attained age 20-30 | | attained age 31-40 | | attained age >=41 | |
|  | RBDR | AER | RBDR | AER | RBDR | AER | RBDR | AER |
| Sex | 2.49 (2.41-2.58) | 206.8 | 4.85 (4.77-4.93) | 678.5 | 4.24 (4.17-4.32) | 959.5 | 3.74 (3.65-3.82) | 1812.2 |
| Man | 3.27 (3.17-3.37) | 304.0 | 6.71 (6.6-6.82) | 1080.5 | 5.1 (5.01-5.2) | 1286.4 | 4.62 (4.53-4.72) | 2173.4 |
| Women |  |  |  |  |  |  |  |  |
| Status at December 2018 (Ending date ) |  |  |  |  |  |  |  |  |
| Alive | 2.05 (1.99-2.1) | 143.3 | 3.7 (3.65-3.76) | 506.4 | 2.98 (2.93-3.03) | 625.0 | 1.94 (1.9-1.99) | 649.2 |
| Death | 62.82 (60.27-65.46) | 6927.5 | 105.49 (103.39-107.63) | 10240.4 | 41.09 (40.23-41.97) | 8140.5 | 22.38 (21.93-22.83) | 9286.1 |
| Year of diagnosis |  |  |  |  |  |  |  |  |
| <1970 | . | . | . | . | 2.42 (2.26-2.58) | 558.2 | 4.06 (4.00-4.16) | 2194.3 |
| 1970-1979 | . | . | 3.26 (3.09-3.43) | 513.7 | 5.52 (5.42-5.62) | 1317.2 | 4.62 (4.51-4.73) | 1819.9 |
| 1980-1989 | 4.61 (4.32-4.91) | 507.1 | 7.33 (7.22-7.43) | 1142.1 | 4.48 (4.39-4.56) | 1051.9 | 4.40 (3.93-4.90) | 1448.7 |
| >=1990 | 2.72 (2.65-2.78) | 233.4 | 4.01 (3.92-4.11) | 529.7 | 2.05 (1.86-2.24) | 296.0 | . | . |
| Age at first cancer |  |  |  |  |  |  |  |  |
| 0-1 | 2.47 (2.38-2.57) | 188.5 | 4.33 (4.2-4.46) | 650.4 | 3.73 (3.59-3.86) | 899.8 | 4.73 (4.53-4.93) | 2381.4 |
| 2-4 | 2.58 (2.47-2.68) | 223.8 | 8.91 (8.74-9.09) | 1419.9 | 4.97 (4.82-5.13) | 1118.5 | 4.17 (4.02-4.34) | 2094.6 |
| 5-9 | 3.46 (3.31-3.61) | 364.1 | 7.06 (6.89-7.22) | 1045.2 | 6.12 (5.98-6.27) | 1454.8 | 3.92 (3.79-4.04) | 1827.8 |
| 10-14 | 4.36 (4.06-4.67) | 475.4 | 3.67 (3.56-3.79) | 488.2 | 4.47 (4.36-4.58) | 1123.2 | 4.84 (4.72-4.95) | 2367.7 |
| ≥15 | . | . | 3.19 (3.06-3.34) | 407.3 | 3.5 (3.38-3.63) | 758.1 | 2.38 (2.25-2.51) | 871.2 |
| First primary cancer type |  |  |  |  |  |  |  |  |
| Other solid cancer | 2.7 (2.42-2.99) | 232.0 | 6.04 (5.77-6.33) | 838.2 | 3.36 (3.14-3.59) | 706.5 | 3.47 (3.21-3.76) | 1369.2 |
| Kidney tumors | 1.95 (1.81-2.1) | 120.2 | 6.46 (6.27-6.65) | 1121.0 | 4.54 (4.4-4.69) | 1095.5 | 4.2 (4.05-4.35) | 1892.1 |
| Neuroblastoma | 1.5 (1.4-1.61) | 68.1 | 4.03 (3.87-4.19) | 559.1 | 2.96 (2.81-3.12) | 602.7 | 8.89 (8.49-9.3) | 4215.7 |
| Lymphoma | 2.32 (2.13-2.53) | 185.5 | 3.38 (3.26-3.5) | 431.4 | 3.83 (3.72-3.94) | 893.3 | 5.07 (4.92-5.22) | 2625.1 |
| Soft tissue sarcomas | 2.43 (2.25-2.62) | 240.6 | 5.63 (5.43-5.84) | 888.6 | 3.73 (3.6-3.88) | 807.1 | 2.5 (2.37-2.63) | 1029.0 |
| Bone sarcomas | 7.84 (7.25-8.46) | 717.6 | 4.55 (4.36-4.74) | 617.7 | 4.6 (4.42-4.79) | 1103.2 | 1.6 (1.5-1.71) | 398.2 |
| Central nervous system tumor | 5.58 (5.34-5.83) | 616.7 | 10.36 (10.09-10.63) | 1506.5 | 9.52 (9.25-9.81) | 2249.7 | 7.58 (7.33-7.84) | 3973.3 |
| Gonadal/Germ cell tumours | 5.36 (4.86-5.89) | 517.7 | 2.47 (2.31-2.64) | 277.6 | 6.16 (5.92-6.41) | 1681.4 | 3.05 (2.85-3.25) | 1371.4 |
| Thyroid tumor | 1.21 (0.58-2.22) | 21.8 | 4.15 (3.58-4.8) | 545.5 | 1.1 (0.75-1.56) | 36.9 | 1.34 (1.16-1.53) | 255.0 |
| Retinoblastoma | 2.49 (2.37-2.62) | 206.9 | 12.03 (11.62-12.46) | 2099.1 | 4.55 (4.11-5.04) | 1161.2 | 2.49 (1.97-3.09) | 745.1 |
| Treatment Received |  |  |  |  |  |  |  |  |
| No radiotherapy or chemotherapy | 1.21 (1.12-1.3) | 30.9 | 2.02 (1.91-2.14) | 187.0 | 1.39 (1.28-1.5) | 121.3 | 1.26 (1.15-1.37) | 181.1 |
| Radiotherapy | 3.45 (3.19-3.74) | 356.7 | 8.15 (7.9-8.41) | 1195.7 | 7.02 (6.82-7.23) | 1829.7 | 3.97 (3.88-4.07) | 2044.4 |
| Chemotherapy | 2.49 (2.4-2.58) | 194.8 | 4.64 (4.54-4.73) | 663.3 | 3.6 (3.51-3.7) | 783.7 | 4.18 (4-4.37) | 2307.3 |
| Radiotherapy and Chemotherapy | 4.56 (4.4-4.71) | 480.8 | 7.49 (7.36-7.62) | 1221.7 | 5.23 (5.14-5.32) | 1290.6 | 5.35 (5.23-5.47) | 2259.0 |
