# Supplementary material for: Long-term hospitalisations in survivors of paediatric solid tumours in France
Source: Sci Rep. 2022 Oct 27;12:18068. doi: 10.1038/s41598-022-22689-w (PMC9613884; doi:10.1038/s41598-022-22689-w)
Supplement: Supplementary file 6 — Supplementary Table 6. [file 41598_2022_22689_MOESM6_ESM.docx]

| Supplementary Table 6. Multivariate analysis of the number of hospitalisation by type of hospitalization. This table shows the risk ratio and 95% of confidence interval. | | | |  |  |  |  |  |  |  |  |  |  |  |  |  |  |  |
| --- | --- | --- | --- | --- | --- | --- | --- | --- | --- | --- | --- | --- | --- | --- | --- | --- | --- | --- |
|  | Infections | Neoplasms | Haematological | Endocrine | Mental | Neurological | Ocular | Auditory† | Cardiovascular | Pulmonary | Gastrointestinal | Skin | Musculoskeletal | Genitourinary | Congenital Malformations | Symptoms Unclassified | Injury - Poisoning | Other Factors |
| Intercept | 0.51 (0.24 -1.11) | 7.32 (6.35 -8.44) *** | 0.55 (0.22 -1.39) | 1.49 (0.93 -2.38) | 0.86 (0.44 -1.66) | 2.96 (1.92 -4.57) *** | 1.63 (0.78 -3.39) | 0.33 (0.07 -1.43) | 3.05 (2.13 -4.37) *** | 0.33 (0.19 -0.57) *** | 0.74 (0.58 -0.95) ** | 1.16 (0.68 -1.97) | 1.78 (1.22 -2.59) *** | 1.81 (1.41 -2.33) *** | 0.78 (0.31 -1.96) | 0.89 (0.61 -1.29) | 0.43 (0.3 -0.62) *** | 4.01 (3.23 -4.97) *** |
| Women (Ref = Men) | 1.14 (0.88 -1.47) | 0.81 (0.78 -0.84) *** | 0.95 (0.73 -1.24) | 0.77 (0.67 -0.88) *** | 1.14 (0.9 -1.45) | 1.06 (0.92 -1.22) | 1.04 (0.83 -1.3) | 0.95 (0.65 -1.39) | 1.19 (1.09 -1.31) *** | 1.38 (1.17 -1.62) *** | 1.02 (0.93 -1.11) | 1.11 (0.9 -1.35) | 1.22 (1.06 -1.39) *** | 0.45 (0.42 -0.49) *** | 0.75 (0.54 -1.04) | 1.03 (0.92 -1.16) | 1.4 (1.23 -1.6) *** | 0.7 (0.65 -0.75) *** |
| Age in 2006 | 1.04 (1 -1.08) ** | 0.95 (0.95 -0.96) *** | 1.01 (0.98 -1.05) | 1 (0.98 -1.02) | 0.96 (0.92 -1) ** | 0.99 (0.97 -1.01) | 0.97 (0.94 -1) | 0.97 (0.92 -1.03) | 0.93 (0.91 -0.94) *** | 1.01 (0.99 -1.04) | 1.01 (1 -1.02) | 1.02 (0.99 -1.05) | 0.99 (0.97 -1.01) | 1.02 (1.01 -1.03) *** | 1.05 (1 -1.1) | 0.99 (0.98 -1.01) | 1.04 (1.02 -1.06) *** | 0.98 (0.97 -0.99) *** |
| Age at first cancer (Ref = 0-1) |  |  |  |  |  |  |  |  |  |  |  |  |  |  |  |  |  |  |
| 2-4 | 1.33 (0.87 -2.02) | 0.9 (0.84 -0.97) *** | 1.26 (0.82 -1.94) | 1.56 (1.2 -2.04) *** | 0.77 (0.53 -1.12) | 1.07 (0.84 -1.38) | 1.45 (1.07 -1.98) ** | 1.57 (0.63 -3.9) | 1.49 (1.26 -1.75) *** | 1.35 (1 -1.84) ** | 1.02 (0.88 -1.17) | 0.66 (0.48 -0.9) *** | 0.92 (0.74 -1.15) | 0.34 (0.31 -0.37) *** | 2.02 (1.1 -3.71) ** | 1.35 (1.11 -1.65) *** | 0.95 (0.77 -1.17) | 1.1 (0.97 -1.24) |
| 5-9 | 0.97 (0.59 -1.59) | 1.17 (1.08 -1.27) *** | 1.86 (1.11 -3.11) ** | 1.66 (1.24 -2.22) *** | 1.44 (0.94 -2.19) | 1.24 (0.94 -1.65) | 1.3 (0.86 -1.95) | 2.88 (1.12 -7.43) ** | 2.12 (1.77 -2.54) *** | 1.74 (1.25 -2.43) *** | 1.11 (0.94 -1.31) | 0.69 (0.48 -1) ** | 0.78 (0.6 -1.01) | 0.36 (0.32 -0.4) *** | 1.89 (0.94 -3.79) | 1.3 (1.03 -1.64) ** | 0.85 (0.66 -1.09) | 1.04 (0.9 -1.21) |
| 10-14 | 0.83 (0.44 -1.58) | 1.59 (1.44 -1.75) *** | 1.23 (0.63 -2.4) | 1.17 (0.81 -1.68) | 1.81 (1.05 -3.12) ** | 0.81 (0.57 -1.15) | 0.8 (0.45 -1.4) | 2.66 (0.87 -8.11) | 1.47 (1.16 -1.86) *** | 1.26 (0.83 -1.93) | 1.06 (0.86 -1.31) | 0.47 (0.29 -0.75) *** | 0.65 (0.46 -0.91) *** | 0.2 (0.17 -0.25) *** | 1.5 (0.65 -3.46) | 1.53 (1.14 -2.05) *** | 0.8 (0.59 -1.09) | 1.2 (1 -1.43) ** |
| ≥15 | 0.49 (0.21 -1.15) | 1.09 (0.96 -1.24) | 0.48 (0.18 -1.28) | 0.95 (0.6 -1.5) | 1.45 (0.7 -3.01) | 0.75 (0.48 -1.18) | 0.85 (0.42 -1.73) | 3.01 (0.8 -11.37) | 1.58 (1.18 -2.11) *** | 1.25 (0.74 -2.1) | 0.82 (0.63 -1.07) | 0.43 (0.23 -0.77) *** | 1.16 (0.78 -1.72) | 0.13 (0.11 -0.17) *** | 0.69 (0.22 -2.18) | 1.17 (0.8 -1.69) | 0.75 (0.52 -1.1) | 1.06 (0.85 -1.34) |
| Year of Diagnosis (Ref = >1990) |  |  |  |  |  |  |  |  |  |  |  |  |  |  |  |  |  |  |
| <1970 | 0.42 (0.11 -1.56) | 1.39 (1.14 -1.7) *** | 1.28 (0.37 -4.39) | 0.66 (0.32 -1.33) | 5.42 (1.57 -18.72) *** | 0.59 (0.29 -1.22) | 1.04 (0.33 -3.31) | 2.76 (0.35 -21.71) | 7.72 (4.86 -12.27) *** | 0.92 (0.4 -2.1) | 1.04 (0.68 -1.6) | 0.9 (0.32 -2.49) | 0.82 (0.42 -1.62) | 0.25 (0.18 -0.35) *** | 0.5 (0.09 -2.76) | 1.17 (0.65 -2.12) | 0.51 (0.27 -0.97) ** | 1.26 (0.87 -1.81) |
| 1970-1979 | 0.91 (0.39 -2.1) | 1.12 (0.98 -1.27) | 1.05 (0.47 -2.37) | 0.86 (0.55 -1.33) | 5.93 (2.64 -13.31) *** | 0.93 (0.59 -1.47) | 1.43 (0.69 -2.96) | 3.63 (1.02 -13.01) ** | 6.19 (4.54 -8.45) *** | 1.69 (1.01 -2.82) ** | 1.06 (0.8 -1.39) | 0.62 (0.32 -1.2) | 1.2 (0.78 -1.85) | 1.04 (0.83 -1.3) | 0.59 (0.2 -1.73) | 1.69 (1.16 -2.47) *** | 0.81 (0.54 -1.21) | 1.18 (0.93 -1.49) |
| 1980-1989 | 1.29 (0.78 -2.14) | 1.12 (1.03 -1.21) *** | 1.2 (0.73 -1.99) | 1.23 (0.93 -1.62) | 2.21 (1.32 -3.69) *** | 1.16 (0.87 -1.53) | 1.39 (0.88 -2.21) | 2.05 (0.92 -4.58) | 3.65 (2.96 -4.51) *** | 1.32 (0.95 -1.83) | 1.2 (1.01 -1.42) ** | 1.07 (0.73 -1.57) | 1.27 (0.97 -1.66) | 1.21 (1.04 -1.4) *** | 1.52 (0.78 -2.96) | 1.24 (0.98 -1.58) | 0.96 (0.75 -1.22) | 1.19 (1.03 -1.38) ** |
| First primary cancer type (Ref = Neuroblastoma) | |  |  |  |  |  |  | 0.22 (0.03 -1.81) |  |  |  |  |  |  |  |  |  |  |
| Other solid cancer | 1.58 (0.79 -3.19) | 1.4 (1.24 -1.58) *** | 1.67 (0.86 -3.25) | 0.74 (0.47 -1.17) | 3.82 (2.18 -6.68) *** | 1.4 (0.95 -2.05) | 1.12 (0.47 -2.7) | 6.05 (2.39 -15.35) *** | 1.35 (1.04 -1.76) ** | 1.91 (1.17 -3.09) *** | 1.95 (1.57 -2.42) *** | 1.97 (1.2 -3.23) *** | 0.65 (0.45 -0.95) ** | 1.45 (1.22 -1.71) *** | 0.24 (0.05 -1.12) | 1.66 (1.21 -2.27) *** | 1.08 (0.76 -1.55) | 1.59 (1.31 -1.93) *** |
| Kidney tumors | 1.63 (1.01 -2.63) ** | 1.06 (0.97 -1.17) | 0.99 (0.61 -1.61) | 0.7 (0.51 -0.97) ** | 1.3 (0.8 -2.11) | 0.5 (0.35 -0.7) *** | 0.75 (0.4 -1.43) | Ref | 0.73 (0.6 -0.88) *** | 1.74 (1.19 -2.54) *** | 1.42 (1.19 -1.7) *** | 0.74 (0.48 -1.14) | 0.78 (0.61 -0.99) ** | 1.22 (1.11 -1.34) *** | 1.18 (0.54 -2.57) | 1.34 (1.05 -1.71) ** | 0.85 (0.65 -1.11) | 1.42 (1.21 -1.65) *** |
| Lymphoma | 1 (0.57 -1.74) | 1.24 (1.12 -1.36) *** | 1.32 (0.77 -2.25) | 0.78 (0.56 -1.09) | 1.14 (0.66 -1.97) | 0.63 (0.45 -0.88) *** | 1.09 (0.57 -2.07) | 1.39 (0.56 -3.45) | 1.2 (0.99 -1.45) | 1.8 (1.21 -2.68) *** | 1.3 (1.07 -1.58) *** | 1.52 (0.99 -2.32) ** | 0.68 (0.52 -0.9) *** | 0.41 (0.36 -0.48) *** | 0.81 (0.33 -1.95) | 1.04 (0.79 -1.36) | 1.07 (0.81 -1.42) | 1.13 (0.96 -1.34) |
| Soft tissue sarcomas | 0.85 (0.48 -1.53) | 1.11 (1.01 -1.23) ** | 0.39 (0.19 -0.82) *** | 0.63 (0.44 -0.91) *** | 2.25 (1.38 -3.67) *** | 0.53 (0.37 -0.78) *** | 1.75 (0.95 -3.2) | 1.88 (0.74 -4.77) | 1.57 (1.3 -1.89) *** | 1.03 (0.66 -1.59) | 1.19 (0.98 -1.45) | 1.59 (1.05 -2.4) ** | 0.67 (0.51 -0.89) *** | 0.57 (0.5 -0.65) *** | 0.96 (0.42 -2.23) | 0.97 (0.74 -1.28) | 1.02 (0.77 -1.35) | 1.28 (1.08 -1.51) *** |
| Bone sarcomas | 1.18 (0.63 -2.22) | 1.54 (1.38 -1.71) *** | 0.81 (0.42 -1.55) | 0.52 (0.34 -0.8) *** | 0.5 (0.23 -1.07) | 0.49 (0.32 -0.77) *** | 0.73 (0.32 -1.68) | 1.35 (0.44 -4.16) | 0.99 (0.79 -1.25) | 0.86 (0.52 -1.42) | 1.01 (0.8 -1.28) | 2 (1.25 -3.22) *** | 1.05 (0.78 -1.41) | 0.35 (0.28 -0.43) *** | 0.4 (0.12 -1.3) | 0.76 (0.55 -1.05) | 1.64 (1.22 -2.19) *** | 1.17 (0.96 -1.42) |
| Central nervous system tumor | 1.28 (0.71 -2.31) | 1.41 (1.28 -1.56) *** | 0.71 (0.41 -1.23) | 2.06 (1.52 -2.79) *** | 2.44 (1.45 -4.11) *** | 3.79 (2.84 -5.06) *** | 2.7 (1.5 -4.87) *** | 5.7 (2.48 -13.07) *** | 0.74 (0.58 -0.93) *** | 2.61 (1.76 -3.87) *** | 1.07 (0.87 -1.32) | 1.13 (0.71 -1.79) | 0.65 (0.48 -0.88) *** | 0.28 (0.23 -0.35) *** | 2.37 (1.17 -4.82) ** | 1.89 (1.45 -2.45) *** | 1.37 (1.03 -1.82) ** | 1.69 (1.43 -1.99) *** |
| Gonadal/Germ cell tumours | 0.79 (0.37 -1.67) | 1.12 (0.99 -1.27) | 0.54 (0.22 -1.37) | 1.53 (1.07 -2.21) ** | 0.97 (0.48 -1.94) | 0.84 (0.55 -1.27) | 0.97 (0.42 -2.26) | 2.29 (0.76 -6.91) | 1.19 (0.92 -1.54) | 0.67 (0.36 -1.26) | 1.53 (1.23 -1.9) *** | 2.26 (1.45 -3.54) *** | 0.59 (0.42 -0.85) *** | 1.36 (1.16 -1.6) *** | 1.75 (0.77 -3.99) | 0.69 (0.47 -1.01) ** | 0.79 (0.55 -1.12) | 1.01 (0.82 -1.24) |
| Thyroid tumor | 1.53 (0.34 -6.82) | 0.43 (0.31 -0.62) *** | 0 (0 -.) | 0.86 (0.31 -2.41) | 6.44 (2.76 -15.02) *** | 1.35 (0.68 -2.69) | 2.27 (0.78 -6.62) | 1.64 (0.19 -14.23) | 0.4 (0.16 -0.97) ** | 1.09 (0.38 -3.14) | 0.98 (0.6 -1.6) | 0.43 (0.06 -3.18) | 1.17 (0.67 -2.06) | 0.84 (0.43 -1.64) | 0.75 (0.16 -3.56) | 1.22 (0.62 -2.38) | 0.76 (0.36 -1.61) | 0.58 (0.32 -1.05) |
| Retinoblastoma | 0.47 (0.2 -1.09) | 1.75 (1.58 -1.94) *** | 1.72 (0.93 -3.2) | 0.57 (0.36 -0.89) *** | 1.27 (0.66 -2.44) | 0.48 (0.31 -0.76) *** | 11.51 (6.82 -19.43) *** | 0.61 (0.12 -3.03) | 0.78 (0.58 -1.03) | 0.81 (0.46 -1.41) | 0.88 (0.7 -1.11) | 0.59 (0.35 -1) ** | 0.37 (0.25 -0.56) *** | 0.38 (0.31 -0.46) *** | 0.61 (0.25 -1.52) | 0.67 (0.46 -0.97) ** | 0.68 (0.48 -0.96) ** | 0.79 (0.64 -0.96) ** |
| Treatment (Ref = No radiotherapy or chemotherapy) | |  |  |  |  |  |  |  |  |  |  |  |  |  |  |  |  |  |
| Chemotherapy | 1.45 (0.88 -2.4) | 1.75 (1.58 -1.94) *** | 3.59 (1.69 -7.66) *** | 1.52 (1.07 -2.17) ** | 0.96 (0.64 -1.43) | 1.27 (0.94 -1.71) | 1.34 (0.85 -2.11) | 1.09 (0.44 -2.7) | 2.56 (1.95 -3.36) *** | 1.17 (0.78 -1.75) | 0.97 (0.83 -1.14) | 1.1 (0.79 -1.54) | 1.08 (0.84 -1.38) | 6.53 (5.34 -7.99) *** | 0.52 (0.28 -1) ** | 1.66 (1.28 -2.16) *** | 1.34 (1.06 -1.69) ** | 0.74 (0.64 -0.85) *** |
| Radiotherapy | 0.71 (0.37 -1.37) | 2.54 (2.28 -2.82) *** | 2.71 (1.2 -6.08) ** | 2.93 (2.05 -4.18) *** | 0.61 (0.38 -0.98) ** | 1.81 (1.34 -2.44) *** | 2.27 (1.42 -3.62) *** | 1.97 (0.86 -4.53) | 2.91 (2.18 -3.88) *** | 2.41 (1.61 -3.61) *** | 1.23 (1.02 -1.47) ** | 1.3 (0.85 -1.99) | 1.45 (1.09 -1.94) *** | 5.35 (4.23 -6.77) *** | 2.96 (1.62 -5.4) *** | 1.62 (1.22 -2.15) *** | 1.27 (0.97 -1.67) | 1.46 (1.25 -1.7) *** |
| Radiotherapyand Chemotherapy | 1.5 (0.92 -2.45) | 3.47 (3.15 -3.83) *** | 4.41 (2.08 -9.38) *** | 3.38 (2.42 -4.72) *** | 1.15 (0.79 -1.68) | 1.29 (0.97 -1.71) | 2.17 (1.41 -3.35) *** | 2.46 (1.12 -5.4) ** | 4.37 (3.36 -5.69) *** | 2.31 (1.59 -3.37) *** | 1.24 (1.06 -1.45) *** | 1.13 (0.8 -1.6) | 1.32 (1.03 -1.68) ** | 9.98 (8.17 -12.19) *** | 0.56 (0.3 -1.03) | 2.16 (1.68 -2.78) *** | 1 (0.79 -1.28) | 1.32 (1.16 -1.51) *** |
| *** p<0.01, ** p<0.05, † Reference in First primary Cancer "Kidney Tumors" | | |  |  |  |  |  |  |  |  |  |  |  |  |  |  |  |  |
