# Supplementary material for: Long-term hospitalisations in survivors of paediatric solid tumours in France
Source: Sci Rep. 2022 Oct 27;12:18068. doi: 10.1038/s41598-022-22689-w (PMC9613884; doi:10.1038/s41598-022-22689-w)
Supplement: Supplementary file 7 — Supplementary Table 7. [file 41598_2022_22689_MOESM7_ESM.docx]

| Supplementary Table 7. Multivariate analysis of the bed-days by types of hospitalizations. This table shows the risk ratio and 95% of confidence interval. | | | |  |  |  |  |  |  |  |  |  |  |  |  |  |  |  |
| --- | --- | --- | --- | --- | --- | --- | --- | --- | --- | --- | --- | --- | --- | --- | --- | --- | --- | --- |
|  | Infections | Neoplasms | Haematological | Endocrine | Mental | Neurological | Ocular | Auditory† | Cardiovascular | Pulmonary | Gastrointestinal | Skin | Musculoskeletal | Genitourinary | Congenital Malformations | Symptoms Unclassified | Injury - Poisoning | Other Factors |
| Intercept | 0.09 (0.06 -0.14) *** | 7.26 (6.45 -8.17) *** | 0.39 (0.27 -0.59) *** | 0.69 (0.51 -0.92) *** | 0.37 (0.24 -0.57) *** | 0.59 (0.45 -0.77) *** | 2.16 (1.17 -3.99) *** | 0.28 (0.08 -0.93) ** | 1.58 (1.3 -1.93) *** | 0.17 (0.13 -0.22) *** | 1.02 (0.87 -1.21) | 0.71 (0.46 -1.09) | 2.6 (2.11 -3.21) *** | 0.23 (0.19 -0.27) *** | 3.65 (2.09 -6.39) *** | 0.54 (0.41 -0.7) *** | 0.43 (0.35 -0.53) *** | 1.03 (0.85 -1.25) |
| Women (Ref = Men) | 0.68 (0.61 -0.77) *** | 0.88 (0.85 -0.9) *** | 1.54 (1.34 -1.78) *** | 0.8 (0.73 -0.87) *** | 2.64 (2.27 -3.06) *** | 1.33 (1.25 -1.41) *** | 0.79 (0.65 -0.96) ** | 0.8 (0.61 -1.04) | 1.83 (1.75 -1.91) *** | 2.09 (1.96 -2.23) *** | 1.23 (1.16 -1.29) *** | 1.01 (0.88 -1.17) | 1.21 (1.13 -1.3) *** | 0.73 (0.7 -0.76) *** | 0.69 (0.57 -0.84) *** | 1.32 (1.22 -1.41) *** | 2.01 (1.89 -2.13) *** | 1.32 (1.25 -1.39) *** |
| Age in 2006 | 1.09 (1.08 -1.11) *** | 0.94 (0.94 -0.95) *** | 1.06 (1.04 -1.08) *** | 1.01 (1 -1.02) | 0.96 (0.94 -0.98) *** | 0.97 (0.96 -0.98) *** | 1 (0.97 -1.03) | 0.92 (0.89 -0.96) *** | 0.94 (0.93 -0.95) *** | 1.05 (1.04 -1.06) *** | 1 (1 -1.01) | 1 (0.98 -1.03) | 0.97 (0.96 -0.98) *** | 0.96 (0.96 -0.97) *** | 0.92 (0.89 -0.95) *** | 0.98 (0.97 -0.99) *** | 1 (0.99 -1.01) | 1.01 (1 -1.02) ** |
| Age at first cancer (Ref = 0-1) |  |  |  |  |  |  |  |  |  |  |  |  |  |  |  |  |  |  |
| 2-4 | 1.33 (1.1 -1.6) *** | 1.04 (0.98 -1.1) | 1.96 (1.53 -2.51) *** | 1.81 (1.53 -2.13) *** | 0.59 (0.45 -0.78) *** | 1.01 (0.9 -1.14) | 1.58 (1.22 -2.03) *** | 4.51 (1.9 -10.69) *** | 3.13 (2.89 -3.4) *** | 1.4 (1.22 -1.6) *** | 0.87 (0.8 -0.94) *** | 0.71 (0.57 -0.89) *** | 0.56 (0.5 -0.64) *** | 2.82 (2.67 -2.97) *** | 1.6 (1.13 -2.28) *** | 2.18 (1.91 -2.48) *** | 1 (0.89 -1.13) | 0.63 (0.58 -0.69) *** |
| 5-9 | 0.52 (0.42 -0.64) *** | 1.24 (1.16 -1.32) *** | 1.56 (1.14 -2.13) *** | 1.94 (1.62 -2.33) *** | 2.14 (1.61 -2.85) *** | 1.7 (1.51 -1.92) *** | 0.57 (0.4 -0.82) *** | 7.43 (3.12 -17.69) *** | 2.46 (2.24 -2.69) *** | 1.72 (1.49 -1.99) *** | 0.88 (0.8 -0.96) *** | 0.72 (0.55 -0.93) *** | 0.73 (0.63 -0.84) *** | 2.13 (1.98 -2.29) *** | 5.61 (3.8 -8.3) *** | 1.37 (1.17 -1.6) *** | 1.37 (1.21 -1.56) *** | 0.76 (0.68 -0.84) *** |
| 10-14 | 0.67 (0.51 -0.88) *** | 1.31 (1.21 -1.42) *** | 0.8 (0.55 -1.17) | 1.42 (1.13 -1.79) *** | 4.17 (2.94 -5.91) *** | 0.99 (0.85 -1.16) | 0.27 (0.15 -0.47) *** | 7.07 (2.72 -18.42) *** | 1.65 (1.48 -1.84) *** | 1.19 (1 -1.41) ** | 0.99 (0.87 -1.11) | 0.6 (0.43 -0.83) *** | 0.98 (0.83 -1.17) | 1.76 (1.59 -1.96) *** | 1.29 (0.75 -2.21) | 1.66 (1.38 -2) *** | 1.41 (1.21 -1.64) *** | 0.75 (0.66 -0.86) *** |
| ≥15 | 0.1 (0.07 -0.15) *** | 1.18 (1.07 -1.3) *** | 0.25 (0.15 -0.42) *** | 1.53 (1.17 -2) *** | 3.14 (1.98 -4.98) *** | 0.9 (0.73 -1.11) | 0.53 (0.28 -1.02) | 5.05 (1.63 -15.6) *** | 1.69 (1.48 -1.94) *** | 0.46 (0.37 -0.57) *** | 0.49 (0.42 -0.58) *** | 0.71 (0.48 -1.06) | 1.29 (1.05 -1.58) *** | 0.53 (0.45 -0.63) *** | 2.42 (0.98 -5.97) | 1.18 (0.93 -1.49) | 1.28 (1.06 -1.54) *** | 0.81 (0.69 -0.95) *** |
| Year of Diagnosis (Ref = >1990) |  |  |  |  |  |  |  |  |  |  |  |  |  |  |  |  |  |  |
| <1970 | 0.1 (0.06 -0.17) *** | 1.38 (1.18 -1.61) *** | 0.15 (0.08 -0.3) *** | 0.91 (0.6 -1.38) | 10.71 (5.07 -22.64) *** | 1.95 (1.4 -2.73) *** | 0.6 (0.21 -1.73) | 24.81 (6.2 -99.35) *** | 6.76 (5.52 -8.28) *** | 0.32 (0.23 -0.44) *** | 1.31 (1.03 -1.68) ** | 1.62 (0.84 -3.14) | 2.87 (2.04 -4.02) *** | 16.56 (13.65 -20.1) *** | 217.92 (73.55 -645.69) *** | 2.47 (1.71 -3.57) *** | 1.46 (1.08 -1.98) *** | 0.66 (0.51 -0.86) *** |
| 1970-1979 | 0.68 (0.48 -0.95) ** | 1.07 (0.97 -1.19) | 0.25 (0.16 -0.39) *** | 1.06 (0.81 -1.38) | 3.81 (2.32 -6.24) *** | 2.49 (2.02 -3.08) *** | 0.75 (0.39 -1.46) | 11.83 (4.8 -29.12) *** | 5.23 (4.55 -6.02) *** | 0.88 (0.72 -1.07) | 1.1 (0.94 -1.3) | 1.02 (0.66 -1.57) | 1.51 (1.2 -1.89) *** | 9.07 (7.94 -10.36) *** | 21.56 (10.18 -45.67) *** | 3.39 (2.67 -4.3) *** | 1.72 (1.42 -2.08) *** | 0.94 (0.8 -1.11) |
| 1980-1989 | 1.13 (0.9 -1.42) | 1.36 (1.27 -1.45) *** | 0.92 (0.71 -1.19) | 1.62 (1.36 -1.92) *** | 3.4 (2.46 -4.69) *** | 2.61 (2.27 -3.01) *** | 1.18 (0.78 -1.8) | 4.31 (2.34 -7.94) *** | 4.14 (3.74 -4.58) *** | 1.5 (1.32 -1.72) *** | 1.05 (0.94 -1.16) | 1.59 (1.22 -2.07) *** | 1.79 (1.55 -2.06) *** | 9.54 (8.69 -10.48) *** | 10.11 (6.22 -16.44) *** | 3.03 (2.6 -3.53) *** | 1.81 (1.61 -2.03) *** | 0.91 (0.82 -1.01) |
| First primary cancer type (Ref = Neuroblastoma) | |  |  |  |  |  |  | 0 (0 -.) |  |  |  |  |  |  |  |  |  |  |
| Other solid cancer | 3.83 (2.58 -5.7) *** | 1.89 (1.71 -2.09) *** | 8.73 (6.45 -11.8) *** | 0.65 (0.5 -0.86) *** | 0.49 (0.3 -0.8) *** | 2.55 (2.08 -3.13) *** | 0.34 (0.08 -1.47) | 8.81 (4.38 -17.7) *** | 1.03 (0.92 -1.16) | 2.07 (1.71 -2.52) *** | 2.16 (1.87 -2.51) *** | 1.2 (0.74 -1.94) | 0.38 (0.29 -0.49) *** | 0.5 (0.43 -0.58) *** | 0.29 (0.13 -0.67) *** | 2.53 (2.03 -3.17) *** | 1.39 (1.17 -1.67) *** | 2.76 (2.37 -3.21) *** |
| Kidney tumors | 5.18 (3.92 -6.84) *** | 1.4 (1.29 -1.52) *** | 1.01 (0.76 -1.33) | 0.72 (0.6 -0.87) *** | 1.07 (0.8 -1.43) | 0.51 (0.42 -0.62) *** | 1.45 (0.84 -2.51) | Ref | 0.37 (0.34 -0.41) *** | 1.53 (1.3 -1.79) *** | 2.09 (1.87 -2.35) *** | 0.92 (0.68 -1.25) | 0.89 (0.78 -1.02) | 0.81 (0.76 -0.86) *** | 0.68 (0.44 -1.07) | 1.6 (1.35 -1.9) *** | 0.55 (0.47 -0.64) *** | 0.99 (0.87 -1.14) |
| Lymphoma | 1.45 (1.06 -1.99) ** | 1.92 (1.77 -2.09) *** | 1.3 (0.97 -1.76) | 0.51 (0.42 -0.63) *** | 0.82 (0.6 -1.13) | 0.9 (0.76 -1.07) | 1.76 (0.99 -3.13) ** | 2.73 (1.43 -5.2) *** | 1.08 (1 -1.18) | 1.44 (1.21 -1.7) *** | 1.42 (1.25 -1.61) *** | 1.4 (1.03 -1.9) ** | 0.63 (0.54 -0.74) *** | 0.4 (0.37 -0.44) *** | 0.69 (0.39 -1.23) | 1.52 (1.26 -1.83) *** | 0.7 (0.6 -0.82) *** | 1.06 (0.92 -1.21) |
| Soft tissue sarcomas | 1.4 (1.01 -1.92) ** | 1.39 (1.28 -1.52) *** | 0.57 (0.37 -0.87) *** | 0.8 (0.66 -0.98) ** | 0.51 (0.36 -0.72) *** | 0.52 (0.42 -0.64) *** | 3.23 (1.91 -5.45) *** | 3.3 (1.63 -6.7) *** | 0.97 (0.89 -1.06) | 0.61 (0.5 -0.74) *** | 2.37 (2.1 -2.68) *** | 1.94 (1.44 -2.61) *** | 0.7 (0.6 -0.82) *** | 0.46 (0.42 -0.5) *** | 0.3 (0.15 -0.58) *** | 1.79 (1.5 -2.15) *** | 0.94 (0.81 -1.1) | 1.52 (1.33 -1.75) *** |
| Bone sarcomas | 5.44 (3.96 -7.47) *** | 2.16 (1.97 -2.37) *** | 1.08 (0.74 -1.58) | 0.4 (0.31 -0.52) *** | 0.07 (0.04 -0.13) *** | 0.47 (0.36 -0.61) *** | 0 (0 -.) | 1.58 (0.61 -4.05) | 0.51 (0.46 -0.58) *** | 1.77 (1.48 -2.12) *** | 0.9 (0.76 -1.06) | 2.62 (1.88 -3.64) *** | 2.59 (2.24 -3) *** | 0.05 (0.04 -0.06) *** | 0.5 (0.21 -1.22) | 1.67 (1.35 -2.06) *** | 2.4 (2.08 -2.78) *** | 1.24 (1.06 -1.44) *** |
| Central nervous system tumor | 2.3 (1.66 -3.2) *** | 2.78 (2.56 -3.01) *** | 0.98 (0.74 -1.32) | 1.77 (1.47 -2.13) *** | 1.58 (1.15 -2.17) *** | 8.53 (7.34 -9.91) *** | 4.06 (2.36 -6.99) *** | 15.93 (8.77 -28.95) *** | 0.71 (0.65 -0.78) *** | 2.38 (2.01 -2.81) *** | 1.5 (1.31 -1.72) *** | 1.72 (1.26 -2.34) *** | 0.69 (0.58 -0.81) *** | 0.49 (0.44 -0.55) *** | 8.61 (5.68 -13.07) *** | 3.85 (3.22 -4.6) *** | 2.7 (2.35 -3.11) *** | 3.64 (3.21 -4.14) *** |
| Gonadal/Germ cell tumours | 1.74 (1.2 -2.53) *** | 2.23 (2.02 -2.46) *** | 1.89 (1.36 -2.64) *** | 1.02 (0.81 -1.27) | 0.25 (0.16 -0.41) *** | 1.31 (1.04 -1.65) ** | 2.15 (1.08 -4.29) ** | 3.18 (1.38 -7.35) *** | 0.72 (0.63 -0.82) *** | 0.19 (0.13 -0.27) *** | 2.79 (2.45 -3.19) *** | 1.67 (1.17 -2.39) *** | 0.56 (0.45 -0.69) *** | 2.59 (2.39 -2.8) *** | 0.67 (0.34 -1.33) | 1.97 (1.6 -2.44) *** | 0.65 (0.54 -0.8) *** | 1.97 (1.69 -2.29) *** |
| Thyroid tumor | 8.3 (4.46 -15.46) *** | 1.79 (1.48 -2.18) *** | 0 (0 -.) | 0.78 (0.44 -1.36) | 1.23 (0.64 -2.34) | 2.29 (1.59 -3.3) *** | 0 (0 -.) | 16.16 (6.18 -42.31) *** | 0.14 (0.09 -0.23) *** | 0.3 (0.16 -0.57) *** | 1.45 (1.07 -1.97) ** | 0 (0 -.) | 1.96 (1.51 -2.53) *** | 0.07 (0.04 -0.13) *** | 0.21 (0.03 -1.54) | 1.03 (0.58 -1.82) | 0.16 (0.08 -0.34) *** | 0.53 (0.31 -0.89) ** |
| Retinoblastoma | 0.68 (0.38 -1.22) | 1.86 (1.71 -2.03) *** | 1.38 (0.95 -1.99) | 0.61 (0.47 -0.81) *** | 0.61 (0.38 -0.97) ** | 1.56 (1.27 -1.91) *** | 8.9 (5.5 -14.42) *** | 0 (0 -.) | 0.27 (0.23 -0.32) *** | 0.78 (0.61 -1) ** | 0.97 (0.82 -1.15) | 0.98 (0.66 -1.45) | 0.44 (0.35 -0.56) *** | 5.55 (5.18 -5.95) *** | 1.32 (0.78 -2.25) | 1.03 (0.81 -1.32) | 0.34 (0.27 -0.43) *** | 3.67 (3.21 -4.19) *** |
| Treatment (Ref = No radiotherapy or chemotherapy | |  |  |  |  |  |  |  |  |  |  |  |  |  |  |  |  |  |
| Chemotherapy | 2.2 (1.69 -2.88) *** | 1.58 (1.45 -1.73) *** | 1.81 (1.39 -2.35) *** | 1.89 (1.53 -2.34) *** | 2.63 (1.92 -3.6) *** | 3.12 (2.48 -3.94) *** | 0.99 (0.68 -1.44) | 0.79 (0.42 -1.5) | 2.86 (2.42 -3.38) *** | 1.93 (1.61 -2.32) *** | 1.32 (1.18 -1.47) *** | 2.72 (2.01 -3.68) *** | 1.47 (1.27 -1.71) *** | 25.56 (22.18 -29.45) *** | 0.72 (0.5 -1.04) | 2.78 (2.32 -3.33) *** | 2.91 (2.5 -3.37) *** | 1.77 (1.54 -2.02) *** |
| Radiotherapy | 0.77 (0.56 -1.06) | 2.87 (2.62 -3.15) *** | 1.3 (0.95 -1.79) | 2.56 (2.07 -3.18) *** | 1.39 (0.99 -1.95) | 6.06 (4.88 -7.53) *** | 1.65 (1.09 -2.49) ** | 1.24 (0.72 -2.15) | 7.82 (6.63 -9.22) *** | 3.69 (3.09 -4.42) *** | 1.18 (1.04 -1.33) *** | 2.59 (1.83 -3.65) *** | 1.49 (1.26 -1.75) *** | 6.25 (5.35 -7.3) *** | 0.99 (0.67 -1.44) | 2.24 (1.85 -2.71) *** | 2.65 (2.26 -3.1) *** | 2.24 (1.96 -2.56) *** |
| Radiotherapy and Chemotherapy | 2.43 (1.87 -3.15) *** | 4.51 (4.14 -4.91) *** | 2.95 (2.26 -3.84) *** | 2.95 (2.41 -3.61) *** | 1.5 (1.09 -2.05) *** | 7.76 (6.26 -9.6) *** | 1.72 (1.2 -2.47) *** | 1.97 (1.15 -3.37) *** | 7.37 (6.27 -8.65) *** | 3.1 (2.61 -3.69) *** | 1.95 (1.76 -2.16) *** | 3.09 (2.28 -4.19) *** | 2.42 (2.09 -2.79) *** | 10.77 (9.33 -12.43) *** | 0.17 (0.11 -0.26) *** | 2.31 (1.93 -2.77) *** | 2.03 (1.75 -2.36) *** | 3.78 (3.34 -4.28) *** |
| *** p<0.01, ** p<0.05, † Reference in First primary Cancer "Kidney Tumors" | | |  |  |  |  |  |  |  |  |  |  |  |  |  |  |  |  |
