# Supplementary material for: Long-term hospitalisations in survivors of paediatric solid tumours in France
Source: Sci Rep. 2022 Oct 27;12:18068. doi: 10.1038/s41598-022-22689-w (PMC9613884; doi:10.1038/s41598-022-22689-w)
Supplement: Supplementary file 8 — Supplementary Table 8. [file 41598_2022_22689_MOESM8_ESM.docx]

| Supplementary Table 8. Characteristics of the studies included in the Discussion. | | | | |  |  |  |  |  |
| --- | --- | --- | --- | --- | --- | --- | --- | --- | --- |
| Autor | Type of CC | Country | N CCSS | Diagnosed | Comparison cohort, N | Hospitalisation follow-up period | Average age at end of folloup | RHR, AER (x10^5^PY) | Hospitalizations studied |
| Kirchhoff (2014) | All | USA | 1,499 | 1973-2005 | 7,219 Comparison cohort based on birth year and sex | 1996-2010 (13.5 years) | 33,12* | Survivors were hospitalized, on average, 1.62 (SD=3.37) not RHR | All excluding pregnancy and delivery |
| Kurt (2011) | All | USA | 10,366 | 1970–1986 | U.S. population rates using age- and sex-stratified standardized incidence ratios (SIRs) | 1992-2005 (13 Years) | Age at 2000 follow-up (years) Mean (SD) 28.6 (7.7) | 1.6 times (95% CI: 1.6; 1.7) / AER of 54.9 hospitalizations per 1,000 person-years. | 11 organ systems/categories: infectious, neoplasm, cardiovascular, pulmonary, psychological, neurologic, gastrointestinal, genitourinary, endocrine, obstetric, and external |
| Sieswerda (2016) | All | Netherlands | 1,382 | 1966-1999 | 26,583 with corresponding year of birth and gender | 1995-2005 (10 Years) | Attained age at the end of follow-up 25.3 (5.9–51.3) | The overall RHR and AER were 2.2 (95%CI:1.9–2.5) and 93.3 per 1000 person-years at risk, | All Hospitalization |
| Streefkerk (2020) | All | Netherlands | 5,650 | 1963-2001 | 109,605 age- and sex-matched controls | 1995-2016 (20 Years) | 30,68* | RHR 2.0 (95% CI: 1.9-2.2) AER was 100.18 per 1,000 PY | Hospitalizations for giving birth were excluded |
| Mueller (2018) | All | USA | 3,152 | 1974–2014 | 31,520 matched on birth year and sex | 1982–2014 Median follow-up after index date was 9.1 years (range 0.1–27.0 years) | 22,58* | Any hospitalization rate (HR 2.7, 95% CI 2.4–3.0) x 1,000 PY not AER reported | Pregnancy-related hospitalizations (ICD9 630–679, 760–779) were excluded |
| De Fine (2017) | All | Nordic Countries | 21,297 | 1943-2008 in Denmark 1971-2008 in Finland 1955-2008 in Iceland 1958-2008 in Sweden | 152,231 matched by age, sex, year, and country | Follow-up was started 5 years after the date of cancer diagnosis or at the beginning of the hospital registers (Denmark, 1977; Finland, 1975; Iceland, 1999; Sweden, from 1968–1987 stepwise inclusion of counties and nationwide since 1987) Follow-up ended on the date of death, the date of emigration, or the end of the study (Iceland: 31 December 2008; Sweden: 31 December 2009; Denmark: 31 October 2010; Finland: 31 December 2012). Mean Follow-up: 16 years; range: 0-42 years | 31,45* | RR of1.94 (95% CI 1.91–1.97).The AER was 3,068 (2,980–3,156) per 100,000 person-years | Not include the ICD sections of ill-defined diseases and the group of injuries and violence in the analysis, Mental disorders , and childbirth and pregnancy complications and congenital malformation or chromosome abnormality (ICD-8 codes 740–759, ICD-10 codes Q00–Q99) |
| Brewster (2014) | All (0-25 Years) | Scotland | 5,229 | 1981 - 2003 | Indirect standardisation for age and sex ompared with the general Scottish population | Follow-up was from 5 years after diagnosis to date of emigration, date of death, or end of 2009 (14,8 Years) | No Info | SHR 2.8 (95% CI 2.7–2.9) AER of 6.4 (6.0–6.6) hospital admissions per 100 cancer survivors per year | Disease-specific outcome codes |
| Font-Gonzalez (2017) | All | Netherlands | 1,382 | 1966 - 1999 | 26,583 corresponding year of birth and gender | 1995 - 2005 (10 years) | Attained age at the end of follow-up 25.3 (19.5–32.1) | The overall RHR and AER for all the four disease groups in CCS compared to the general population were 7.2 (95% CI: 5.5–9.4) and 38 per 1000 person-years at risk, respectively. | four categories neoplasms, diseases of the circulatory system, endocrine/ nutritional/metabolic diseases and diseases of the eye. |
| Lorenzi (2011) | All | Canada | 1,374 | 1981-1995 | 13740 frequency matching was done by gender and birth year | 1986 - 2000 Mean follow-up time for survivors was 7 years (maximum 15 years). | No Info | Relative risk of hospital-related late morbidity (4.1 (3.7–4.5)) | except pregnancy |
| * Estimated from reported data | | |  |  |  |  |  |  |  |
