# Supplementary figures and images for: Long-term hospitalisations in survivors of paediatric solid tumours in France
Source: Sci Rep. 2022 Oct 27;12:18068. doi: 10.1038/s41598-022-22689-w (PMC9613884; doi:10.1038/s41598-022-22689-w)

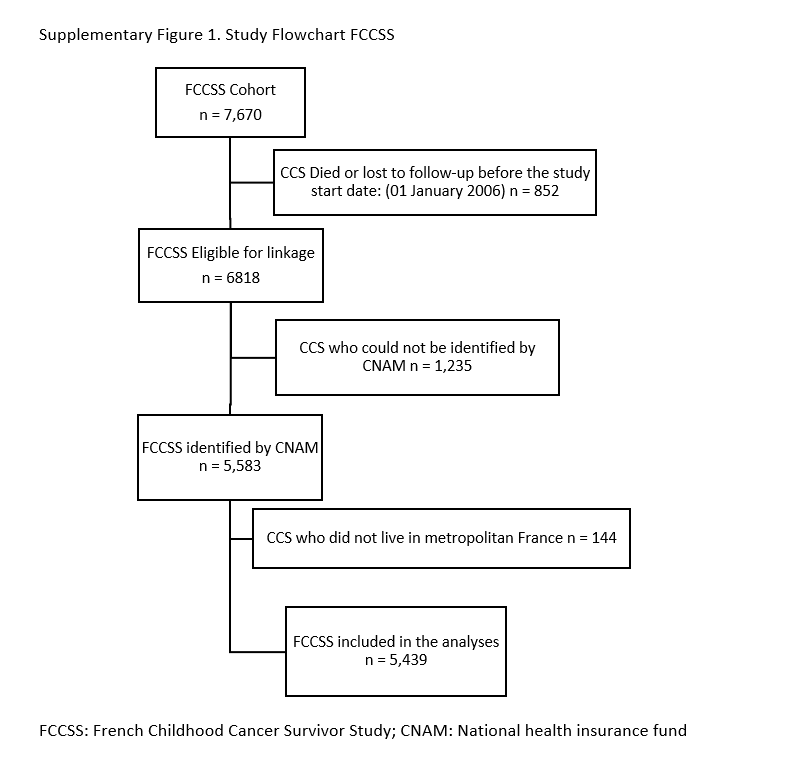

Supplement: Supplementary file 9 — Supplementary Figure 1. [file 41598_2022_22689_MOESM9_ESM.tif]
